# Clinical isolates of *Providencia rettgeri* and *Providencia Stuartii* evades neutrophil-mediated killing by subverting neutrophil-extracellular traps

**DOI:** 10.3389/fimmu.2025.1636387

**Published:** 2025-10-02

**Authors:** Joselyn E. Castro, Federico Birnberg-Weiss, María J. Toloza, Yasmín A. Bertinat, Federico Fuentes, Agustín Peyloubet, Diego Faccone, Sonia A. Gómez, Verónica I. Landoni, Gabriela C. Fernández

**Affiliations:** ^1^ Laboratorio de Fisiología de los Procesos Inflamatorios, Instituto de Medicina Experimental (IMEX)-CONICET/Academia Nacional de Medicina de Buenos Aires, Ciudad Autónoma de Buenos Aires, Argentina; ^2^ Laboratorio de Genética Hematológica, Instituto de Medicina Experimental (IMEX)-CONICET/Academia Nacional de Medicina de Buenos Aires, Ciudad Autónoma de Buenos Aires, Argentina; ^3^ Instituto de Medicina Experimental (IMEX)-CONICET/Academia Nacional de Medicina de Buenos Aires, Ciudad Autónoma de Buenos Aires, Argentina; ^4^ Servicio de Antimicrobianos, Instituto Nacional de Enfermedades Infecciosas (INEI), ANLIS ‘Dr. Carlos G. Malbrán’, Ciudad Autónoma de Buenos Aires, Argentina; ^5^ Consejo Nacional de Investigaciones Científicas y Técnicas (CONICET), Argentina

**Keywords:** neutrophils, immune evasion, NETs, nucleases, *Providencia rettgeri*, *Providencia stuartii*

## Abstract

**Introduction:**

*Providencia rettgeri* (Pr) and *Providencia stuartii* (Ps) are clinically relevant opportunistic pathogens. They are resistant to several antibiotics including carbapenems. The immune response against these pathogens has never been investigated. Here, we aimed to evaluate whether neutrophils (PMN), key players against bacterial infections, were able to recognize and eliminate these bacteria.

**Methods:**

We measured PMN functions after challenge with selected clinical isolates of Pr and Ps, and used *Escherichia coli* ATCC (Eco), which fully activates PMN, for comparison. Bacterial survival was evaluated after exposure of PMN to bacteria for 1 or 3 h and colony formation units (CFU) were determined.

**Results:**

While PMN were able to partially contain Ps growth at 1 h, at 3 h both Pr and Ps were able to escape PMN-mediated killing compared to Eco, which was efficiently killed. Reactive oxygen species (ROS) generation was not induced by Pr or poorly induced by Ps, compared to Eco, but phagocytosis of Pr, Ps, and Eco was similar. Although Pr and Ps induced the release of double-stranded (d.s.) DNA early at 30 min (vital neutrophils extracellular traps or NETs), the release of late-induced NETs (3 h, suicidal NETs) was not observed, consistent with the absence of PMN death observed with Pr or Ps. In addition, Pr and Ps decreased suicidal NETs when Eco or PMA were used as inducers. This decrease was abolished by fixed bacteria, and was dependent on the release of a DNase activity. Twenty-four h after i.p. inoculation of mice with Pr, Ps or Eco, all bacteria induced migration of PMN to the peritoneum, but no PMN activation or NETs was observed in Pr or Ps-treated mice. When the distribution of bacteria in different organs was measured by CFU determination, Pr and Ps disseminated to the spleen and lungs, whereas Eco was exclusively present in the peritoneum.

**Discussion:**

The isolates used in this study of Pr and Ps are poor inducers of bactericidal PMN responses and display immune evasion strategies to subvert PMN-mediated killing. These evasion mechanisms, acting on degrading vital NETs and/or blocking the formation of suicidal NETs, would favor bacterial dissemination.

## Introduction

The misuse of antimicrobials has accelerated the emergence of multi-resistant (MDR) bacterial strains, making infectious diseases increasingly difficult to treat. Approximately 700,000 people die each year due to infections with MDR microorganisms, and by 2050, the number of deaths from this cause would rise to >8 million, exceeding the number of deaths from cancer (1). Invasive procedures like catheter placement and surgery, and inadequate infection control practices, such as poor hand hygiene and environmental cleaning, among others, can facilitate the spread of MDR bacteria within the Intensive Care Units (ICU). Infections with these microorganisms are difficult to treat and have high economic costs, mainly because they generate prolonged stays, require greater use of medications, and require more laboratory and other studies for diagnostic purposes.

*Providencia* spp. includes the *P. alcalifaciens*, *P. rettgeri, P. stuartii, P. rustigianii* and *P. heimbachae*. They are commonly found in soil, water, and wastewater (2). Only *P. stuartii* and *P. rettgeri* are clinically relevant in humans, and nowadays are widely recognized as notorious opportunistic pathogens capable of causing a wide variety of nosocomial infections (2, 3). Urinary tract infections are the most common infections caused by these bacteria, followed by bacteremia, pneumonia, meningitis, endocarditis, and wound infections (2). Infections with *P. stuartii* and *P. rettgeri* have a significant impact on morbidity, mortality, and treatment of infected patients (4, 5). This is due, in part, since *P. stuartii* and *P. rettgeri* are resistant to gentamicin, tobramycin, aminopenicillins, and first generation cephalosporins (2), can produce AmpC-type β-lactamases, and possess intrinsic resistance to colistin and tigecycline (6). Furthermore, recent reports indicate that nosocomial *P. stuartii* and *P. rettgeri* have acquired plasmids carrying genes that encode carabapenemases of the serine enzyme type, such as KPC (*Klebsiella Pneumoniae* Carbapenemase) or metallo-beta-lactamase, such as NDM (New Delhi metallo-beta-lactamase) (7, 8). These enzymes hydrolyze carbapenems, the last-resort antibiotics. Reports of carbapenemase-resistant *P. stuartii* and *P. rettgeri* have been increasing alarmingly (9), being described for the first time in Japan in 2003 (10), and later detected in other countries such as Argentina, Brazil, Canada, China, Ecuador, Greece, India, Israel, Italy, Mexico, Portugal, South Africa, South Korea, England and the United States, among others (11). For patients with co-morbidities or immunosuppression, the emergence of multidrug-resistant strains is extremely dangerous, given the fragility of their immune status and the lack of effective responses for their treatment. Further complicating this situation, another important characteristic of opportunistic bacteria is their ability to evade the immune system. In this sense, they have evolved different mechanisms to avoid detection and destruction by the host’s immune response. Studying the host-pathogen interaction is crucial to understand the relevant mechanisms necessary to overcome an infection. In this sense, to date, no studies have been published on the immune response against the clinically relevant species of *Providencia* spp.

Neutrophils (PMN) are the first line of defense against bacteria, and they are rapidly recruited from circulation to sites of microbial invasion by host and pathogen-derived stimuli. They have different microbicidal mechanisms to combat infections, including intracellular and extracellular mechanisms. After reaching the infectious focus, PMN recognize bacteria and engulf them in phagosomes. Once inside the phagosome, production of toxic reactive oxygen species (ROS) by the assembly of a multiprotein complex called NADPH oxidase, and the fusion of different types of granules that contain bactericidal proteins with phagosomes are the main intracellular mechanisms that mediate bacterial death. Together with this, the release of web-like structures composed of chromatin fibers called neutrophil extracellular traps (NETs) can trap microbes, preventing their spread to other tissues, and can directly kill bacteria or inhibit their growth (12). Two types of NETs have been described in response to a microbial challenge. Vital NETs are rapidly induced, and are independent on reactive oxygen species (ROS) production (13). Moreover, during vital NETs formation, PMN remain viable retaining their membrane integrity, and some of their normal functions, such as their ability to phagocytose and migrate (14). Different studies have reported that, depending on the stimulus, the source of DNA released by this mechanism could be of mitochondrial or nuclear, in the latter case via nuclear blebbing and vesicular exportation (14). On the other hand, suicidal NETs initiate later after activation, depend on ROS, and result in cell death. This programmed cell death pathway, different from apoptosis or necrosis, is characterized by nuclear membrane disassembly, chromatin decondensation, and subsequent NETs release after plasmatic membrane permeabilization. In suicidal NETs, cytoplasmic and granule proteins, such as elastase, are dragged together with chromatin, providing this type of NETs with greater bactericidal potential. Recent works point to the generation of NETs as a fundamental mechanism to contain the spread of bacteria in urinary infections (15, 16).

Considering all this, our aim was to evaluate whether PMN can mount an effective microbicidal response upon challenge with selected clinical isolates of *P. stuartii* and *P. rettgeri*, or if, on the contrary, bacterial evasion mechanisms are taking place to favor their survival. It is essential to understand the biology of infections with multi-resistant organisms to design different and/or alternative strategies to the use of antibiotics, aimed at improving the immune response against this type of pathogens.

## Materials and methods

### Bacterial isolates used and susceptibility testing

All experiments were performed using a local clinical isolate of *Providencia stuartii* (Ps, M21250) and *Providencia rettgeri* (Pr, M17517), and *Escherichia coli* ATCC^®^ 25922™ (Eco) as a positive control. Other clinical isolates of Pr (n=4) and Ps (n=4) were included for studying some of the parameters, and were obtained from patients of different hospitals from Buenos Aires between April 2013 to September 2017 (See table in **Supplementary Material 1** for details of the isolates used). All Pr or Ps strains were different from each other according to their pulse field gel electrophoresis (PFGE) pattern. Bacterial identification was performed by typical biochemical methods and by Maldi TOF (Bruker, London, UK). Susceptibility testing was determined by disc diffusion following protocols and interpretation criteria established by CLSI.

### Bacterial preparation and growth conditions

The strains were cryopreserved at -80 °C. For use, they were seeded in tryptic soy agar (TSA) and incubated at 35 ± 2 °C for 18 h to obtain isolated colonies. Then, a single colony was inoculated in tryptic soy broth (TSB) and incubated at 35 ± 2 °C for 24 h in static conditions. After that, 100 µL of the culture was added into 10 mL of fresh TSB, and grown for another 4 h with agitation, until reaching log phase. Bacteria were pelleted by centrifugation at 9600 x g for 15 min, washed twice in phosphate buffered saline (PBS) 1 x and resuspended at the desired concentration in PBS 1 x. Bacteria concentration was determined by measuring O.D. at 600 nm, and adjusting to 0.09 absorbance units, that is equivalent to 1 x 10^8^ colony forming units (CFU)/mL. CFU concentration was confirmed by counting on TSA plates. Bacteria were prepared at the desire working concentration in RPMI 1640 supplemented with 2% of heat-inactivated fetal calf serum (FSC). All experiments were performed using a ratio PMN:bacteria of 1:10 unless otherwise specified.

### Blood samples and ethics statement

Blood samples were obtained from healthy volunteer donors who had not taken any medication for at least 10 days before the day of sampling. Blood was obtained by venipuncture of the forearm vein and was drawn directly into citrated plastic tubes. This study was performed according to institutional guidelines (National Academy of Medicine, Buenos Aires, Argentina) and received the approval of the institutional ethics committee (N° 19/22/CEIANM), and written informed consent was provided by all the subjects.

### Polymorphonuclear neutrophil isolation

PMN were isolated by Ficoll-Hypaque gradient centrifugation (Ficoll Pharmacia, Uppsala; Hypaque, Wintthrop Products, Buenos Aires, Argentina) and dextran sedimentation, as previously described (17). Contaminating erythrocytes were removed by hypotonic lysis. After washing, the cells (96% PMN on May Grünwald/Giemsa-stained Cyto-preps) were suspended in RPMI 1640 supplemented with 2% FCS and used immediately after. All experiments where PMN were used were performed in RPMI 1640 supplemented with 2% FCS.

### Bactericidal activity assays

Bacteria alone or bacteria in the presence of PMN in a 1:1 ratio were left for 1 h or 3 h at 37 °C, 5% CO_2._ After incubation, serial dilutions in H_2_O were performed for each sample, (bacteria grown alone for 1 or 3 h or bacteria incubated with PMN for 1 or 3 h) and the resultant solutions were plated in duplicates on TSA. CFU were counted the following day, and CFU were normalized using the following equation: CFU (normalized) = $\frac{\text{CFU }+\text{ PMN}}{\text{CFU }-\text{ PMN}}$ , where CFU + PMN was the number of CFU obtained for bacteria incubated for 1 or 3 h in the presence of PMN, and CFU - PMN was the number of CFU obtained for each bacterium grown for 1 or 3 h in the absence of PMN.

### Chemotaxis

Chemotaxis was quantified using a modification of the Boyden chamber technique (18). PMN (1 x 10^5^) in 50 µL were placed in triplicate in the top wells of a 48-well micro-chemotaxis chamber. A PVP-free polycarbonate membrane (3 µm pore size; Neuro Probe Inc. Gaithersburg MD, USA) separated the cells from lower wells containing either RPMI or the different bacteria (1x10^6^). The chamber was incubated for 30 min at 37 °C in a 5% CO_2_ humidified atmosphere. After incubation, the filter was stained with TINCION-15 (Biopur SRL, Rosario, Argentina), and the number of PMN on the undersurface of the filter was counted in a five random high-power fields (HPF) x 400 for each of triplicate.

### FSC and CD11b expression determination

PMN (5 x 10^5^) were incubated with the different bacterial strains in a PMN:bacteria ratio of 1:10 for 30 min at 37 °C in a 5% CO_2_ humidified atmosphere. Then, a specific mouse anti-human CD11b antibody conjugated with phycoerythrin (PE) (Biolegend, San Diego, CA, USA Cat. 301306) was added, and cells were incubated in the dark at 4 °C for 30 min. The FSC and the mean fluorescence intensity of CD11b were determined on 50,000 events by flow cytometry after excluding debris by FSC-SSC using a FACSCanto (Becton Dickinson). The gating strategy used to exclude doublets is depicted in **Supplementary Material 2**. The increase in the percentage of FSC and the CD11b mean fluorescence intensity (MFI) was analyze on single cells. For CD11b results were normalized according to the following formula: MFI CD11b (normalized) = $\frac{\text{MFI CD}11\text{b of the different treatments }}{\text{MFI CD}11\text{b of untreated PMN }\left(-\right)}$ .

### Phagocytosis

PMN was challenged with bacteria in a 1:10 ratio for 1 h at 37 °C in 5% CO_2_. Then cells were fixed, and processed for observation by transmission electron microscopy (TEM). The number of PMN with bacteria inside was quantified in 100 cells, and expressed as the percentage of phagocytosis. The number of bacteria inside individual PMN was also registered and expressed as the frequency of PMN with different number of bacteria (# bacteria/PMN).

### Reactive oxygen species generation

ROS production was measured by flow cytometry (FACSCanto, Becton Dickinson) using Dihydrorhodamine 123 (DHR, Sigma Aldrich). PMN (5 x 10^5^) were incubated with 1 μM of DHR for 15 min at 37 °C. Subsequently, cells were incubated with or without the different bacterial strains for 30 min at 37 °C 5% CO_2_ in a humidified atmosphere. Immediately after, the H_2_O_2_ production was determined in the FL-1 channel.

### Confocal microscopy determination of neutrophil extracellular traps

NETs by confocal microscopy were determined as previously reported (19). Briefly, PMN (5 x 10^5^) were seeded gently onto glass coverslips coated with 0.001% poly-L-lysine in a 24-well plate in triplicate, allowed to settle, and incubated in the presence of bacteria. Cells were incubated for 3 h at 37 °C, 5% CO_2_. After the incubation period, samples were gently fixed with 4% paraformaldehyde (PFA) for 15 min at room temperature. After fixation, samples were washed three times with PBS 1x and incubated in a blocking buffer (PBS 1 x, 3% BSA) for 1 h at room temperature. Then, a primary rabbit anti-neutrophil elastase antibody (Merk KGaA, Darmstadt, Germany; Cat. 481001) was added and incubated for 1 h at room temperature. After washing with PBS 1 x three times, samples were incubated with a donkey anti-rabbit IgG secondary antibody conjugated with Dy-Light 649 (Biolegend, San Diego, CA, USA; Cat. 406406) for 1 hour at room temperature in the dark. After washing, samples were treated with triton x100 (0.25%) for 15 min, and were mounted with Vectashield mounting medium containing propidium iodide (Vector Laboratories, Inc. Burlingame, CA, USA; Cat H-1300). Images for NETs evaluation were acquired using a FluoView FV1000 confocal microscope (Olympus, Tokyo, Japan) equipped with a Plapon 60 x/1·42 objective lens and processed using Olympus. At least 10 different fields were observed in each triplicate (200 x). NETs areas were determined as previously reported in at least 5 microphotographs obtained in 200 x for each sample (20), using the wand tool of the FIJI software (21). The scale for the measurement was obtained from the data given in the confocal microscope image. To measure nuclear decondensation, PMN were incubated for 90 min with the different bacteria and samples were fixed, permeabilized with triton x 100 (0.25%) for 15 min and stained using the same protocol detailed above. Microphotographs were obtained in 600 x. The number of PMN with decondensed nucleus were quantified in at least 100 cells and expressed as a percentage.

### Extracellular double strand DNA measurement

PMN (1 x 10^6^) were incubated in the presence of the different bacteria for time indicated in each experiment at 37 °C, 5% CO_2_. After the incubation period, a commercial micrococcal Nuclease S7 (4 U, Roche, Basilea, Suiza) was added for 15 min at 37 °C in order to cut and release the DNA associated with NETs from the cell body of PMN. After inactivation of the enzyme by the addition of 5 mM of EDTA, supernatants were collected and centrifuged twice, first at 900 x g and then at 9600 x g to eliminate bacteria. The presence of double stranded (d.s.) DNA was quantified in duplicates using the commercial kit Quant-iT™ PicoGreen^®^ (Invitrogen, Thermo Fisher, Waltham. MA. USA) that contains a DNA intercalator, which was read in a fluorimeter (DeNovix DS-11 spectrophotometer).

### Quantitative PCR for nuclear or mitochondrial gene determination in NETs

PMN (1 x 10^6^) were incubated with Pr or Ps in PMN:bacteria ratio of 1:10 for 60 min at 37 °C, 5% CO_2_ for vital NETs induction. After the incubation period, NETs were cut from the PMN body as detailed above and a total of 250 µL of this supernatant was used for DNA extraction with the Bosphore Nucleic Acid Extraction Versatile Spin Kit (Anatolia Geneworks, Istanbul, Turkey), following manufacturer’s instructions. Subsequently, 30 ng of the extracted DNA was used in consecutive quantitative PCR (qPCR) reactions, each with a final volume of 20 µL. Reactions included 10 µM of specific primers targeting the actin for nuclear DNA determination (actin-FW 5´- atgtttgagaccttcaacacccc-3´, actin-RV 5´- gccatctcttgctcgaagtccag-3´) or ND1 for mitochondrial DNA detection (ND1-FW 5´-aacatacccatggccaacct-3´, ND1-RV 5´agcgaaqgggttgtagtagccc-3´). All qPCR assays were performed using the SsoAdvanced SYBR Green Supermix (2×) and run in the CFX96 Dx Real-Time PCR Detection System from Bio-Rad (San Francisco, CA, USA). Results were expressed as the fold increase using the following formula: Fold increase = $\frac{1}{\text{Ct normalized}}$ , where Ct normalized = $\frac{\text{Ct forPr or Ps }}{\text{Ct for untreated PMN }\left(-\right)}$ .

### PMN death

PMN (5 x 10^5^) were challenged with bacteria in a 1:10 ratio for 3 h at 37 °C in 5% CO_2_. After the incubation period, propidium iodide (PI, 1 µg/mL) was added for 15 min to assess the loss of plasmatic membrane integrity (cell death) and the percentage of PI positive cells were determined by flow cytometry. In another set of experiments, the release of lactate dehydrogenase (LDH) was determined in PMN incubated with the different bacteria in a 1:10 ratio for 3 h at 37 °C in 5% CO_2_. After, the incubation period, samples were centrifuged, and the supernatants were used for LDH determination in duplicates using the LDH-L kit (Wiener Lab., Argentina) following manufacturer’s instructions.

### NETs formation in co-incubation conditions

PMN were co-incubated with Pr o Ps and PMA (40 ng/mL) or Eco (ratio 1:1, Eco: PMN) at 37 °C, 5% CO_2_. After 3 h, NETs area or d.s. DNA was determined as stated above. In some experiments, Ps and Pr were fixed with PFA (4%, 30 min), or bacterial supernatants (sn) were obtained from bacterial cultures grown in RPMI 1640 for 4 h, filtered using a 0.22 µm filter and then supplemented with 2% FCS. PMN in RPMI 2% FSC were then incubated with fixed bacteria or sn (1:1, vol/vol), and NETs formation assays using PMA were performed.

### DNase activity

To assess the DNase activity of Pr and Ps or of bacterial sn, purified eukaryotic DNA (600 ng/mL) was incubated with bacteria or sn for 1 h at 37 °C adding Ca2_+_ (50 mM) and Mg2_+_ (50 mM), and then d.s. DNA was evaluated using the commercial kit Quant-iT™ PicoGreen^®^ (Invitrogen, Waltham, MA, USA) or an agarose gel was performed to visualized DNA. A commercial nuclease S7 (Nuc S7) (Roche, Basilea, Suiza) was used as a positive control. To reverse DNase activity, samples were treated with EDTA (50 mM).

### Whole genome sequencing and analysis

Genomic DNA was extracted using the QIACube DNAMini Kit (Qiagen, Germantown, MD, USA). WGS was performed on Ps M21250 and Pr M17517 using the Nextera XT DNA library preparation kit and the Illumina MiSeq Platform (Illumina, San Diego, CA, USA). FastQC and Trim Galore were used for quality assessment and trimming. Reads were *de novo* assembled using Spades and confirmed for species with Kraken. Automated annotation was performed with Prokka. Average nucleotide identity (ANI) values were determined using the ANI calculator and ANI clustermap tool. Genome analysis was performed using the Comprehensive Genome Analysis tool offered by Patric 3.6.8 and with the ANLIS-Malbrán analysis pipeline, including ARIBA (MLST and resfinder) and AMRfinderPlus. Other bioinformatics tools were used to search for or compare specific genes or amino acid sequences of capsule and nucleases, such as BLAST and UniProt, using the appropriate reference for each species (search words: nuclease, endonuclease, and exonuclease).

### *In vivo* experiments

BALB/c mice were bred in the animal facility of IMEX-CONICET. Male mice aged 9–16 weeks and weighing 20–25 g were used. They were maintained under a 12-hlight–dark cycle at 22 ± 2 °C and fed with standard diet and water *ad libitum*. The experiments performed were conducted according to the principles set forth in the *Guide for the Care and Use of Laboratory Animals* (National Research Council, 1996). Bacterial strains were prepared as stated above, and 1 x 10^7^ CFU/mice were inoculated by intraperitoneal (i.p.) injection. After 24 h, mice were sacrifice and the peritoneal liquid (pl) was collected. PMN were counted with turk´s solution using an optical microscope. MPO was determined incubating 40 µL of the pl with 50 µL of the specific commercial substrate 3, 3’, 5, 5’-tetramethyl-benzidine (1 x TMB) (Invitrogen, Thermo Fisher, Ealtham, MA, USA) for 15 min at room temperature in the dark. After the incubation period, the reaction was stopped using 30 µL of H_2_SO_4_ (1 M), measuring the resultant absorbance at 450 nm, subtracting the absorbance at 570 nm using a microplate reader (Varioskan™ LUX, Thermo Scientific). Released d.s. DNA was measured as detailed above. The spleen and lungs were also obtained and processed to obtain single cell suspensions. To estimate dissemination from the inoculation site, CFU in the lung, spleen or pl recovered at 24 h were determined by serial dilution in TSA plates, and expressed as the relative bacterial distribution, calculated as follows: Relative bacterial distribution (%) = $\frac{\text{CFU recovered from the lung or spleen or pl}}{\text{total CFU recovered }\left(\text{lung}+\text{spleen}+\text{pl}\right)\;}$ x100.

### Statistical analysis

Statistical differences were determined using the Prism 5.0 software (Graph Pad Software, La Jolla, CA) after applying the Shapiro-Wilk normality test. When normality was determined, the one-way analysis of variance (ANOVA) followed by the Tukey´s post-test for multiple comparisons was performed. Otherwise, the non-parametric Kruskal-Wallis test followed by the Dunn´s post-test for multiple comparisons was performed. P values less than 0.05 were considered significant, and the exact p values are shown in the graphs. The eta-squared (η²) size effect of the ANOVAs performed were calculated as the sum of squares for the effect divided by the total sum of squares. This statistical parameter together with the confidence intervals of the comparisons made for each figure are included in the **Supplementary Material 3**.

## Results

### Selected isolates of *Providencia rettgeri* and *Providencia stuartii e*scape from PMN-mediated killing

**Figure 1:**
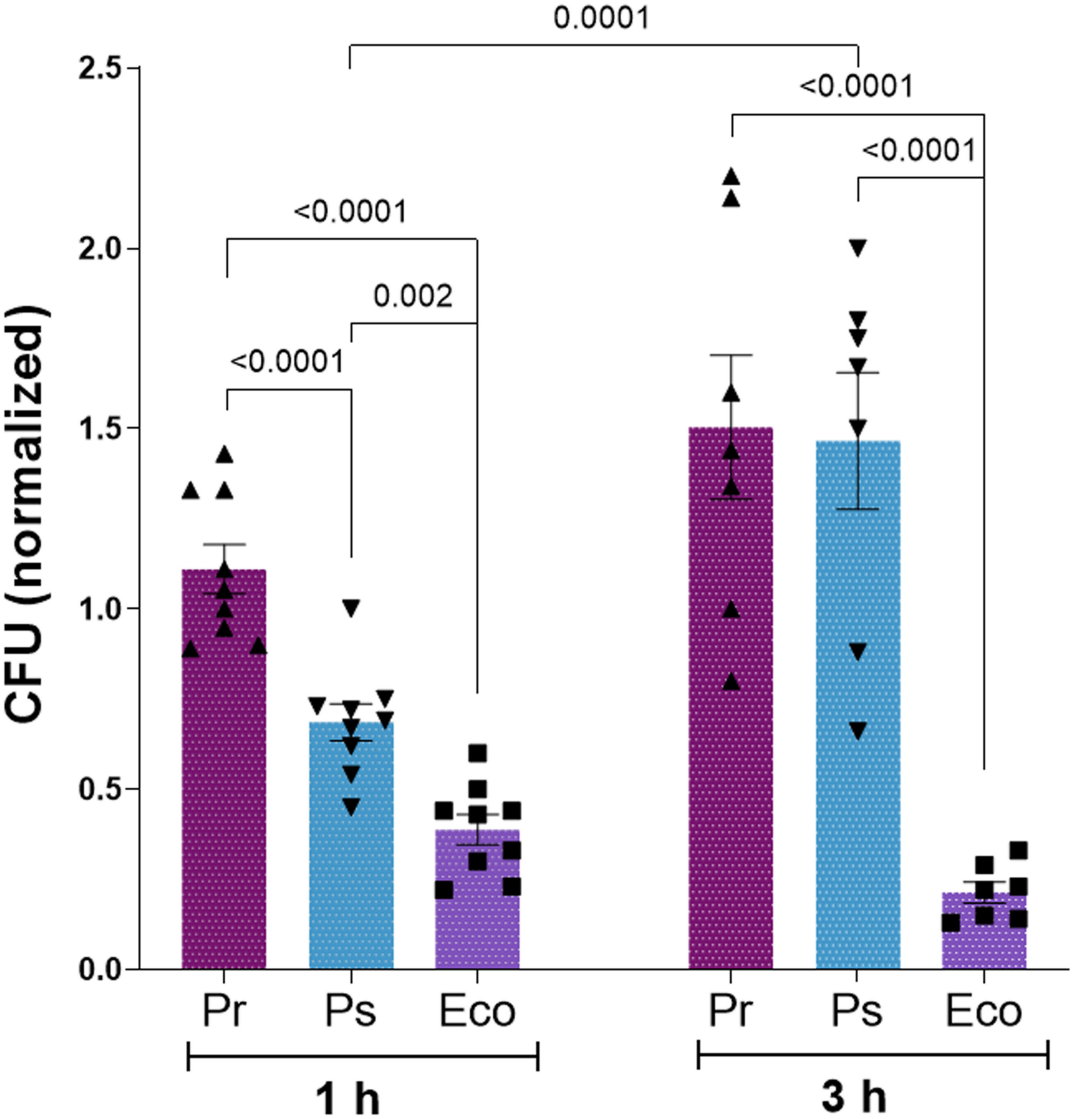
Pr and Ps subvert PMN-mediated killing. Pr, Ps or Eco were left alone (CFU – PMN) or were incubated in the presence of PMN in a bacteria:PMN ratio of 1:1 (CFU + PMN) for 1 or 3 h. Colony forming units (CFU) were determined in each sample and values normalized as described in Materials and Methods. All results were expressed as the mean ± SEM; each point represents a different PMN donor. Data presented a normal distribution according to the Shapiro-Wilk normality test, and comparisons were performed using one-way ANOVA followed by the Tukey´s post-test for multiple comparisons.

In this study, we selected one clinical isolate of *Providencia rettgeri* (from now on termed as Pr) and one of *Providencia stuartii* (from now on termed as Ps) to study how PMN respond to challenges with these isolates. As a positive control, we used an *Escherichia coli* ATCC (Eco) strain that has been used in previous studies and fully activates PMN (22, 23). We determined whether PMN could efficiently eliminate Pr or Ps performing killing assays. For this purpose, Pr, Ps, or Eco were left alone or were confronted with PMN for 1 h or 3 h, and total CFU were determined and expressed as described in Material and Methods. As depicted in **Figure 1**, 1 h after challenge, Eco and Ps were partially eliminated, whereas Pr was not. However, at 3 h post-challenge, Ps increased its survival, reaching values similar to those obtained for Pr. These results indicate that even though Ps is partially contained by PMN at early time points, both Pr and Ps finally subvert PMN-mediated killing, evidencing immune evasion mechanism that allow them to survive.

### Pr and Ps induce chemotaxis and PMN activation

**Figure 2:**
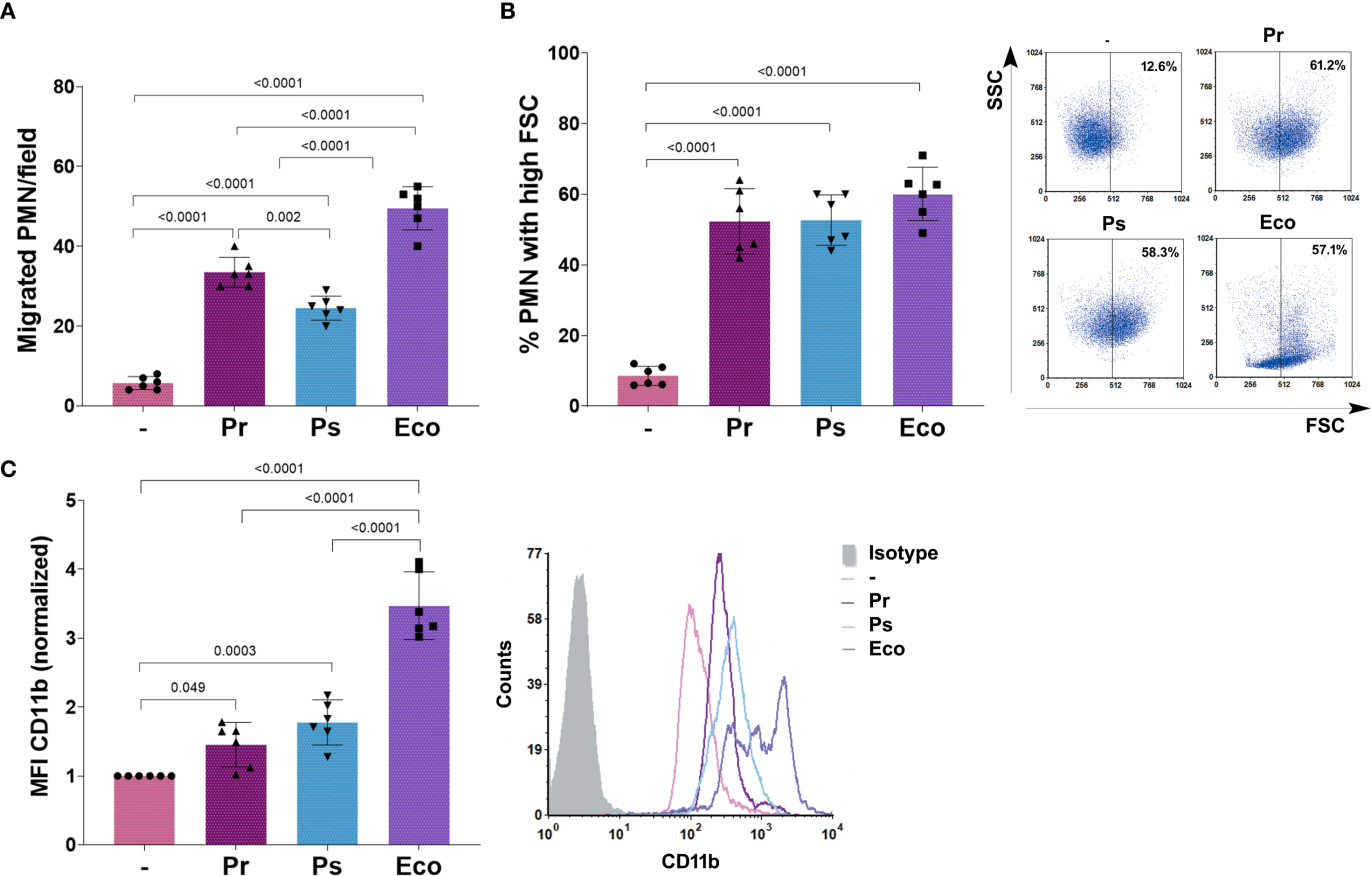
Pr and Ps are recognized by PMN. Pr, Ps or Eco were incubated with PMN in a bacteria:PMN ratio of 10:1 for 30 min. All results were expressed as the mean ± SEM; each point represents a different PMN donor. In all cases, data presented a normal distribution according to the Shapiro-Wilk normality test, and comparisons were performed using one-way ANOVA followed by the Tukey´s post-test for multiple comparisons. **(A)** Number of PMN/field that migrated toward the chemotactic stimulus (bacteria). **(B)** % of PMN that increased their Forward Scatter (FSC) determined by flow cytometry. Right panel: representative dot-plots showing SSC versus FSC profiles of PMN without treatment (–), Pr, Ps, or Eco. **(C)** Expression of CD11b determined by flow cytometry. The values of the mean fluorescence intensity (MFI) of CD11b were normalized to the values obtained for unstimulated PMN (–) as described in Materials and Methods. Right panel: Representative histograms of the expression of CD11b in the different experimental groups.

To study what PMN responses might be related to the ability of Pr and Ps to survive in the presence of PMN, we first analyzed the capacity of PMN to migrate towards the bacterial strains using a chemotaxis chamber. Medium alone or the different bacteria were used as the chemotactic stimulus. PMN were allowed to migrate across a membrane, and migrated PMN were quantified. As observed in **Figure 2A**, PMN migrated towards Pr, Ps, and Eco compared to medium alone, but chemotaxis towards Ps and Pr was less effective than towards Eco. Moreover, Ps was less chemotactic than Pr.

Activation of PMN causes changes in cell size, reflected as an increase in the forward scatter (FSC), and the up-regulation of surface molecules, such as CD11b, that mediate endothelial binding necessary to transmigrate to the site of infection. To determine if Pr or Ps activated PMN, we measured the percentage of PMN with a high FSC after bacterial challenge. We found that Pr, Ps, and Eco were similarly able to cause an increase in the % of PMN with high FSC (**Figure 2B**). As can be observed in the representative dot-plots, Eco also affected the side scatter (SSC) profile, whereas Pr and Ps did not. Moreover, when we measured the changes in CD11b expression, we found that compared with untreated cells, all bacteria caused a statistically significant increase in the expression of CD11b. However, both Pr and Ps caused a lower up-regulation compared to Eco (**Figure 2C**).

These results indicate that both Pr and Ps can be initially recognized by PMN, migrating towards bacteria and becoming activated by them. However, the induced response is less robust than the one observed with Eco, which fully activates PMN.

To determine whether the PMN responses induced by Pr and Ps chosen for this study were representative of other clinical isolates, we determined the effect of Pr and Ps on CD11b expression using 4 other isolates of Pr and Ps (see **Supplementary Material 1**, **Supplementary Material 4A**). Although in some cases particular isolate presented slight differences compared to the others, the effect of Pr and Ps on CD11b expression with the pooled isolates was similar to that seen for the chosen isolates.

### Pr and Ps are phagocytosed by PMN but induce a poor respiratory burst

**Figure 3:**
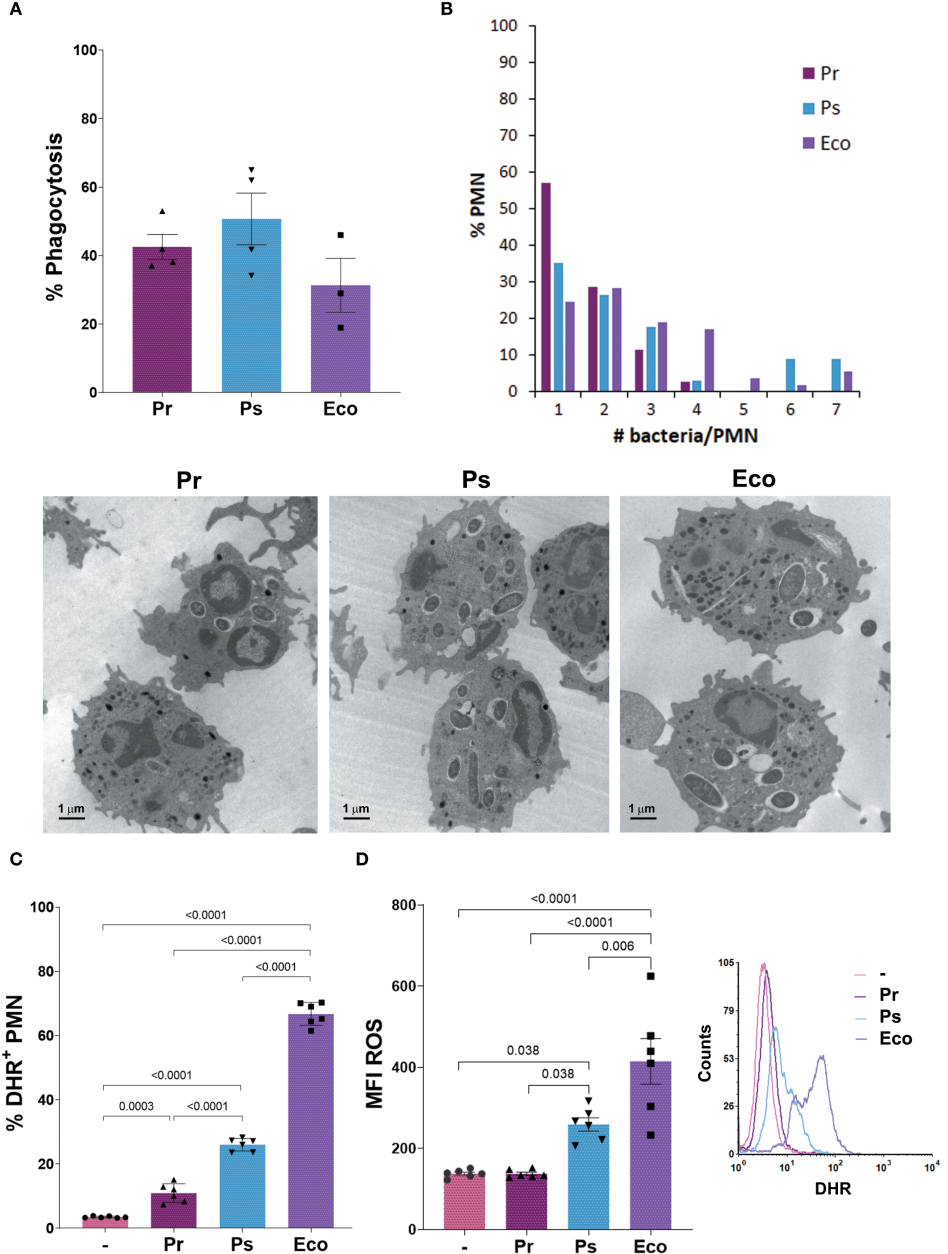
Pr and Ps are phagocytosed by PMN but induced a poor respiratory burst. Bacteria were incubated with PMN in a bacteria:PMN ratio of 10:1. All results were expressed as the mean ± SEM; each point represents a different PMN donor. In all cases, data presented a normal distribution according to the Shapiro-Wilk normality test, and comparisons were performed using one-way ANOVA followed by the Tukey´s post-test for multiple comparisons. **(A)** The percentage of phagocytosis was assessed at 1 h by transmission electron microscopy (TEM). **(B)** The number of bacteria phagocytosed *per* PMN was quantified from the TEM pictures and expressed as the percentage of PMN with the different number of bacteria inside. Lower panel: Representative photographs. **(C)** The percentage of DHR+ PMN (ROS-producing PMN) was measured at 30 min, using dihydrorhodamine (DHR) by flow cytometry. **(D)** Amount of ROS produced *per* PMN (MFI ROS). Right panel: Representative histogram showing the Counts versus DHR of the different experimental groups.

After initial contact with bacteria, PMN must ingest microorganisms to kill them intracellularly by triggering the production of reactive oxygen species (ROS). In this sense, we first evaluated whether Pr and Ps were phagocytosed by PMN. For this, we measured the percentage of PMN with ingested bacteria by transmission electron microscopy (TEM) after 1 h of incubation. As **Figure 3A** shows, the percentage of PMN with intracellular bacteria (% Phagocytosis) was similar for Pr, Ps, and Eco. However, when the number of bacteria inside PMN was counted, we found that the percentage of PMN with<2 bacteria *per* PMN predominates for Pr, but for Ps and Eco PMN with 6 or 7 bacteria were also observed (**Figure 3B**), indicating that even if the % of PMN that ingested bacteria was similar for all groups, less Pr was internalized compared to Ps or Eco.

When the percentage of ROS-producing PMN was evaluated, all bacterial strains induced a statistically significant increase compared to unstimulated PMN. However, the % induced by Pr was lower compared to Ps and Eco, and the % induced by Ps was lower compared to Eco (**Figure 3C**). When the amount of ROS *per* PMN (MFI ROS) was determined (**Figure 3D**), Pr showed no differences compared to unstimulated PMN. Although the amount of ROS induced by Ps was higher compared to untreated PMN, it was lower than that observed for Eco. These results show that Pr and Ps are poor ROS inducers, being Pr even less stimulatory than Ps, and these differences could be associated with the differences in the number of bacteria phagocytosed by PMN.

When we determined the % of ROS producing PMN with 4 other isolates of Pr and Ps (**Supplementary Material 4B**), we found that the pooled samples showed the same response pattern as the one found with the isolates used throughout this study.

### Pr and Ps failed to induce suicidal neutrophil extracellular traps

**Figure 4:**
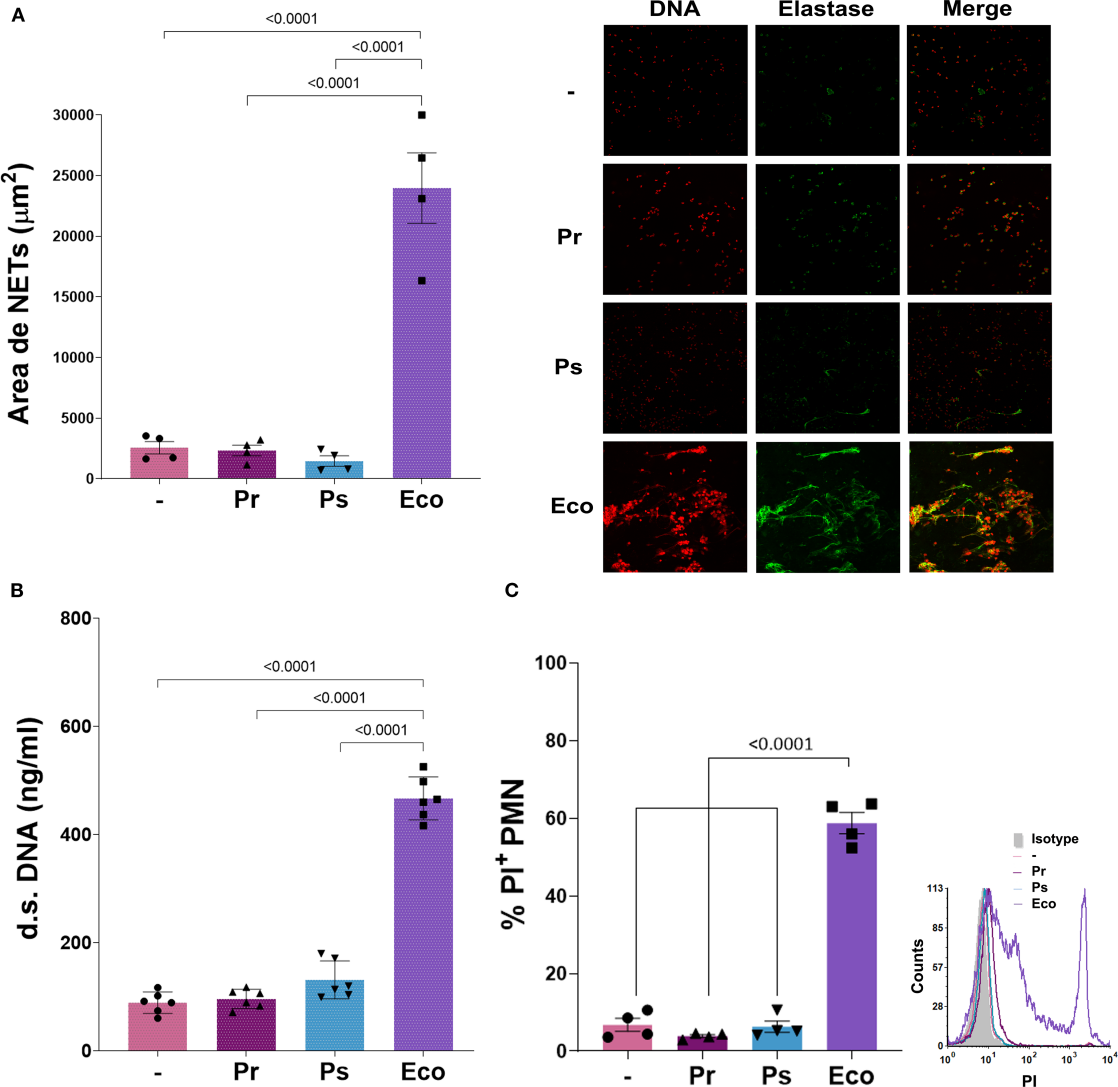
Suicidal Neutrophil extracellular traps (NETs) are not triggered by Pr or Ps in human PMN. Isolated PMN were incubated with Pr, Ps, or Eco in a bacteria:PMN ratio of 10:1 for 3 h The formation of NETs was determined by confocal microscopy and double-stranded (d.s.) DNA release. All results were expressed as the mean ± SEM; each point represents a different PMN donor. In all cases, data presented a normal distribution according to the Shapiro-Wilk normality test, and comparisons were performed using one-way ANOVA followed by the Tukey´s post-test for multiple comparisons. **(A)** NETs area (μm^2^) determined by confocal microscopy using propidium iodide (PI) and a specific antibody, for DNA and elastase staining, respectively. Double-positive DNA-elastase fibers were considered NETs for area quantification. Right panel: Representative microphotograph of NETs (200 x). **(B)** d.s. DNA release. **(C)** PMN viability determined by propidium iodide (PI) staining. The percentage of PI^+^ PMN was assessed by flow cytometry. Right panel: Representative histogram of PI fluorescence intensity for the different experimental groups.

Suicidal NETs release is one of the main mechanisms of extracellular PMN-mediated killing. Together with other granular proteins, elastase co-localizes with DNA fibers after NETs release. To determine suicidal NETs formation, we measured NETs area of released DNA positive for propidium iodide (PI) and elastase by confocal microscopy, and also quantified double-strand (d.s.) DNA released from NETs after 3 h of bacterial challenge. As observed in **Figures 4A, B**, no NETs were found when PMN were challenged with Pr and Ps compared to Eco, which was a strong inducer of NETs release. Reinforcing this result and taking into account that the release of suicidal NETs leads to cell death, we found that after 3 h of incubation with the different bacterial strains, Pr and Ps did not cause PMN death as measured by the lack of propidium iodide (PI) internalization by flow cytometry, whereas Eco induced a high percentage of PI+ PMN (**Figure 4C**). Additionally, cell death was also assessed by the release of LDH in PMN after 3 h of incubation with the different bacteria, and no LDH release was observed by Pr and Ps (LDH (AU) (–): = 0.285 ± 0.005; Pr = 0.300 ± 0.010; Ps = 0.295 ± 0.005; Eco = 0.705 ± 0.025*, n=3, *p<0.0001 vs (–).). The absence of NETs was not associated with a lack of stimulus, as increasing the bacteria:PMN ratio did not lead to an increase in NETs formation (**Supplementary Material 5A**). Moreover, to investigate if factors present in human serum (HS) could reverse the absence of NETs observed with Pr or Ps, these assays were performed in 50% of HS but no increase in d.s. DNA was observed in these conditions either (**Supplementary Material 5B**).

In line with the results shown in previous sections for other parameters, when PMN challenged for 3 h with 4 other isolates of Pr or Ps, the same results were found as with the chosen isolates and no d.s. DNA release was observed (**Supplementary Material 4C**).

These results indicate that suicidal NETs are not observed and PMN remain viable when challenged with Pr or Ps.

### Pr and Ps affect NETs formation triggered by other stimuli

**Figure 5:**
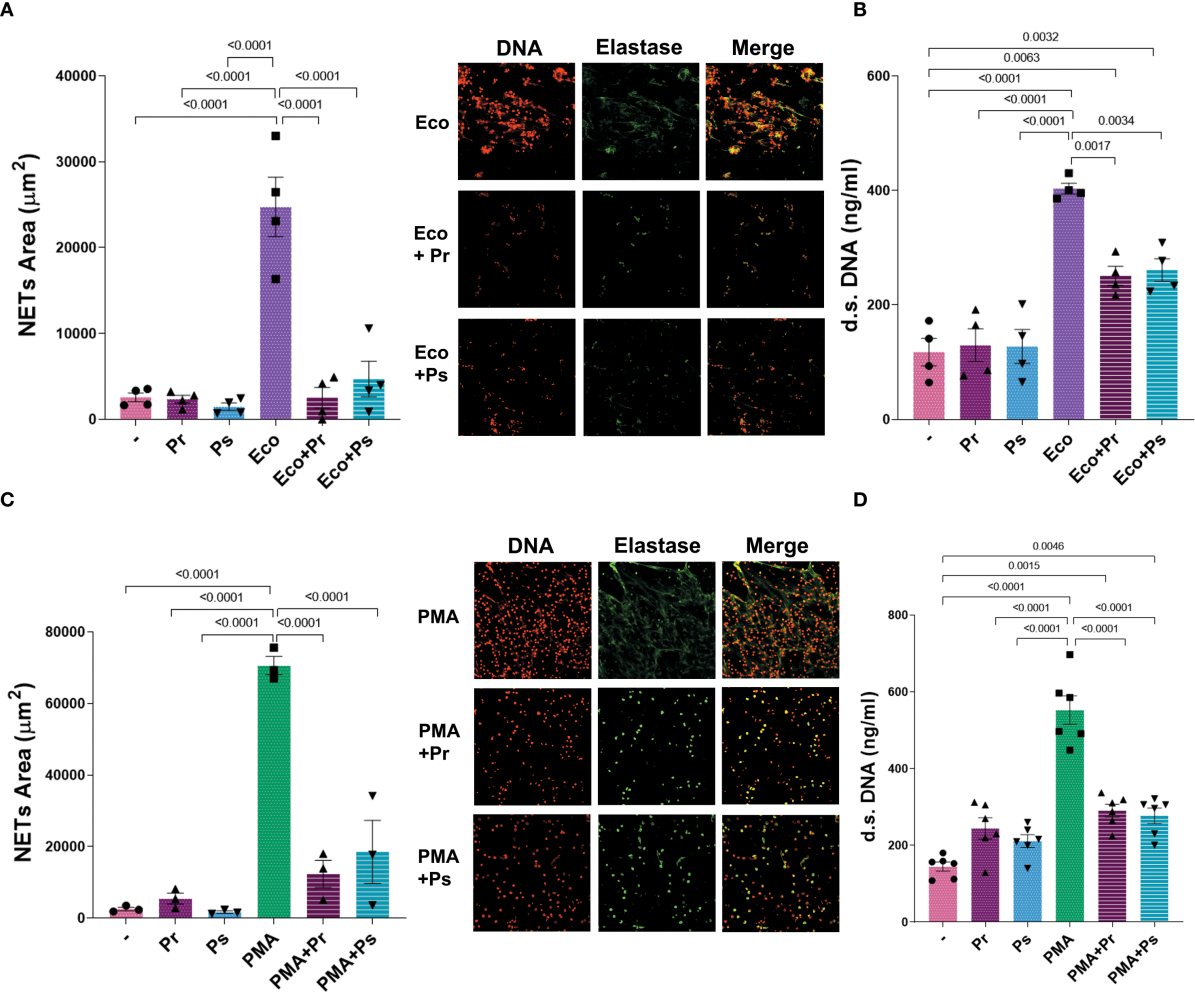
Pr and Ps affect suicidal NETs formation triggered by other stimuli. NETs were evaluated in PMN after being challenged with the different stimuli for 3 h. All results were expressed as the mean ± SEM; each point represents a different PMN donor. In all cases, data presented a normal distribution according to the Shapiro-Wilk normality test, and comparisons were performed using one-way ANOVA followed by the Tukey´s post-test for multiple comparisons. **(A)** NETs area (µm2) with representative microphotographs in the right (200 x), and **(B)** d.s. DNA release in the presence of Eco 1:1 ± Ps or Pr 10:1. **(C)** NETs area (µm^2^) with representative microphotographs in the right (200 x), and **(D)** d.s. DNA release in the presence of PMA (40 nM) ± Ps or Pr 10:1.

Up to this point, Pr and Ps can induce some degree of activation in PMN, but NETs formation seems to be the most affected PMN function. Therefore, we next asked whether, in addition to not being induced, Pr and Ps could affect the formation of NETs induced by other stimuli. For this purpose, Pr or Ps were confronted with PMN stimulated with Eco or the well-known NET-inducer PMA. As shown in **Figure 5**, both Pr and Ps decreased NETs area and d.s. DNA triggered by Eco (**Figures 5A, B**) or PMA (**Figures 5C, D**). These results indicate that Pr and Ps affect NETs triggered by different stimuli, evidencing the presence of NETs evasion strategies that interferes with this type of extracellular-killing mechanism.

### Pr and Ps release a DNase

**Figure 6:**
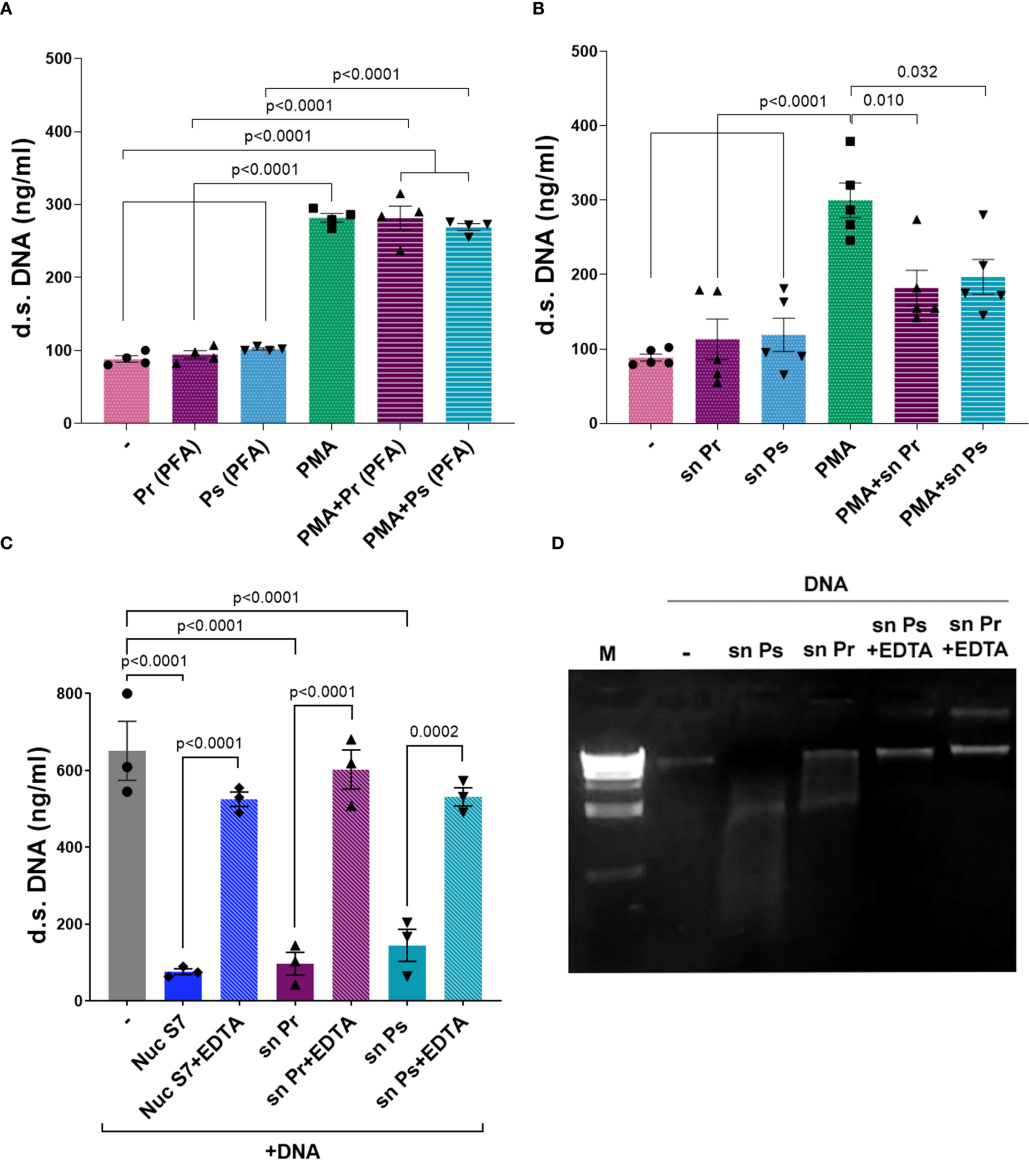
Pr and Ps viability is necessary for decreasing suicidal NETs triggered by PMA and depends on a DNase released by bacteria. Ps and Pr were fixed with PFA, or bacterial supernatant (sn) were collected, and were incubated with PMN ± PMA for 3 h. All results were expressed as the mean ± SEM; each point represents a different PMN donor. In all cases, data presented a normal distribution according to the Shapiro-Wilk normality test, and comparisons were performed using one-way ANOVA followed by the Tukey´s post-test for multiple comparisons. **(A)** d.s. DNA release in the presence of PMA (40 nM) ± Ps (PFA) or Pr (PFA) 10:1. **(B)** d.s. DNA release in the presence of PMA (40 nM) ± Ps (sn) or Pr (sn). **(C)** d.s. DNA quantification of sn obtained from Ps and Pr incubated for 1 h with eukaryotic DNA (600 ng/mL) ± EDTA (50 mM). **(D)** Visualization of eukaryotic DNA degradation by Pr or Ps sn and reversion by EDTA in a 2% agarose gel stained with ethidium bromide.

To assess if the decrease of NETs in response to PMA caused by Pr and Ps was related to bacterial viability, Pr and Ps were fixed, and dead bacteria were used together with PMA to determine NETs formation. As observed in **Figure 6A**, dead Pr or Ps could not decrease NETs release triggered by PMA, indicating that bacterial viability is necessary to reduce NETs. Moreover, when cell-free supernatants (sn) obtained from Pr or Ps cultures were used in the presence of PMA, we found that these sn decreased d.s. DNA induced by PMA (**Figure 6B**). This decrease was not cause by the release of toxic factors that may cause PMN death, as PMN viability was not affected in PMN incubated for 3 h with Pr- or Ps-derived sn (% of PI^+^ PMN: Untreated PMN (–) = 3.18 ± 0.34; sn Pr = 2.38 ± 0.20; sn Ps = 2.520 ± 0.03; n=3). These results indicate that live Pr and Ps actively release a factor into the supernatant that can decrease NETs in response to PMA.

A common strategy to subvert NETs-mediated killing is to affect any of the components that comprise NETs. In this sense, one well-known mechanism used by pathogens is to degrade the NET (24). Considering the above finding, we studied whether Pr and Ps release DNases into the sn that degrade the DNA-fibers of NETs. For this purpose, we incubated isolated eukaryotic DNA with filtered sn obtained from bacterial cultures and used a Nuclease S7 (Nuc S7) for comparison. Besides, according to the requirement of DNases to use bivalent cations for optimal activity, we evaluated the impact of EDTA, a bivalent cation chelator. As depicted in **Figures 6C, D**, the sn of Pr and Ps were able to degrade eukaryotic DNA. Moreover, DNA degradation was reversed by the addition of EDTA. As a complementary analysis of these findings, genomic DNA was extracted and whole gene sequencing was performed on Pr and Ps and their sequences were submitted to GenBank under BioProject PRJNA1304938. The analysis of these sequences resulted in the identification of different genes encoding endo- or exo-nucleases in Pr and Ps, which were included in **Supplementary Material 1**.

These results indicate that Pr and Ps can release DNases to the extracellular medium that may degrade the eukaryotic DNA released in NETs.

### Pr and Ps do not cause nuclear decondensation and induce the release of vital NETs

**Figure 7:**
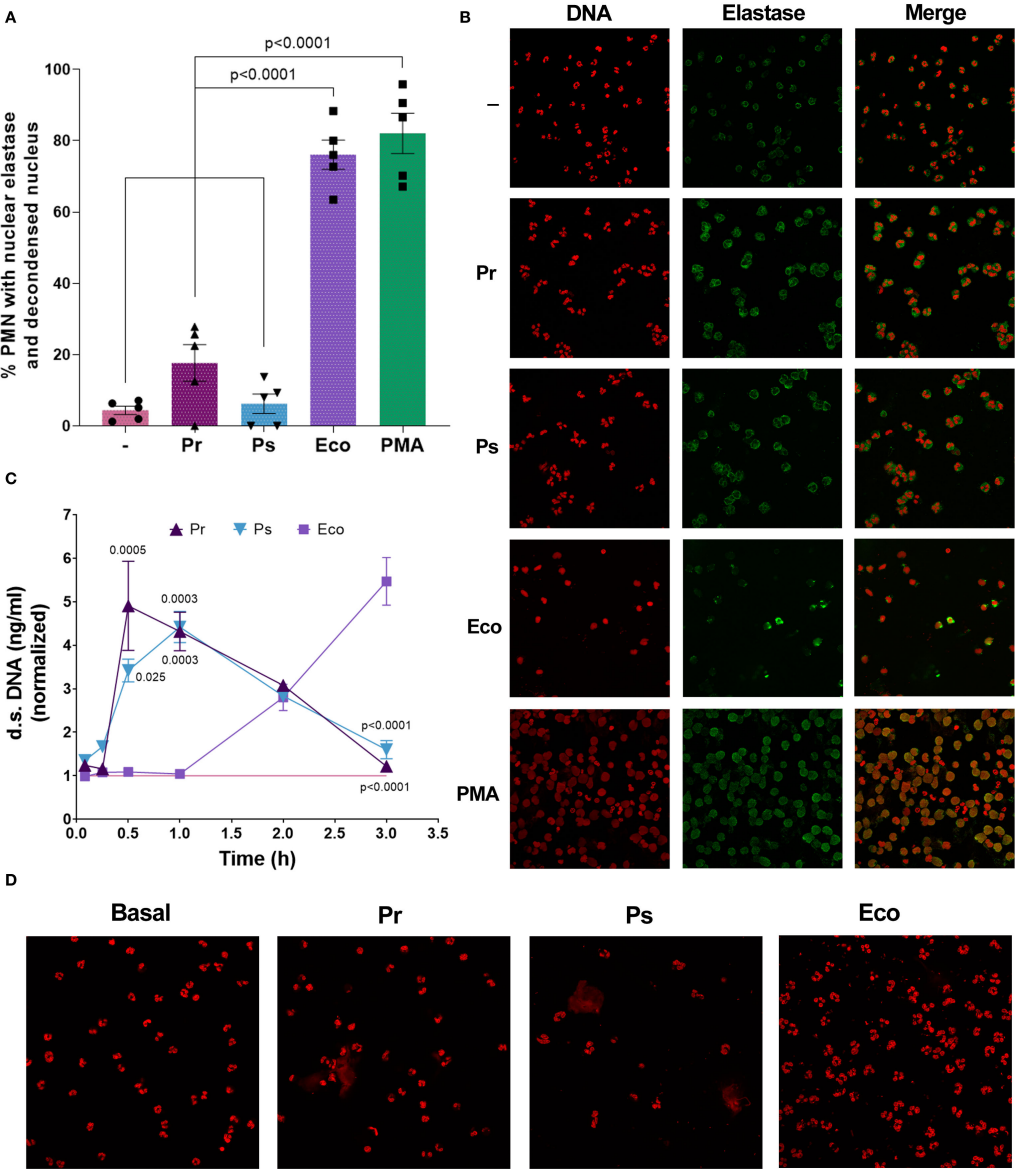
No nuclear elastase with DNA decondensation was observed in PMN challenged with Pr and Ps but vital NETs were induced by bacteria. **(A)** Percentage of PMN with nuclear DNA decondensation co-localizing with elastase quantified by confocal microscopy after incubation with Pr, Ps, Eco, or PMA for 90 min in a bacteria:PMN ratio of 10:1. All results were expressed as the mean ± SEM; each point represents a different PMN donor. Data presented a normal distribution according to the Shapiro-Wilk normality test, and comparisons were performed using one-way ANOVA followed by Tukey´s post-test for multiple comparisons. **(B)** Representative microphotographs (600 x) from **(A)** showing individual DNA and elastase staining and the resulting superposition (merge). **(C)** Extracellular d.s. DNA release from PMN challenged with Pr, Ps, or Eco in a bacteria:PMN ratio of 10:1 measured at different time points. The values were normalized to those obtained for untreated PMN (–) (pink line). Results were expressed as the mean ± SEM (n=4–8 for each time point). Data for each time point presented a normal distribution according to the Shapiro-Wilk normality test and was analyzed using one-way ANOVA followed by Tukey´s post-test for multiple comparisons. The p values refer to comparisons vs. Eco. **(D)** Representative microphotographs (600 x) of confocal microscopy experiments of PMN challenged for 30 min with Pr, Ps, or Eco, showing DNA stained with PI.

In light of the results presented above, revealing the presence of DNases in the sn of Pr and Ps, we wondered whether the lack of NETs at 3 h could be due not to a lack of induction but to an underdetection caused by their degradation. In this sense, the presence of decondensed DNA co-localizing with elastase in PMN nuclei would indicate that NETs release had occurred, but was not detected because of degradation by bacterial DNases. Conversely, if PMN nuclei possess intact non-decondensed DNA, this would confirm the lack of induction of suicidal NETs formation. To clarify this issue, and considering that the release of suicidal NETs is preceded by nuclear decondensation and migration of elastase to the nucleus, we determine the number of PMN with decondensed nuclei and nuclear elastase by confocal microscopy 90 min after bacterial challenge. As depicted in **Figures 7A, B**, neither Pr nor Ps increased the percentage of PMN with nuclear decondensation co-localizing with elastase, whereas Eco and PMA did. This result reinforces our previous observation indicating the lack of suicidal NETs induction by Pr and Ps.

In view of the potential role that DNases could have in the context of immune evasion, and as the early induction of another type of NETs, called vital NETs, has been reported for other bacteria, we next investigated the possibility that Pr or Ps were inducers of this mechanism. For this purpose, PMN were challenged with bacteria and extracellular d.s. DNA was measured between 5 min and 3 h. As shown in **Figure 7C**, we found that both Pr and Ps induced a statistically significant release of d.s. DNA, detected early at 30 min, compatible with vital NETs. This NETs remained high at 1 h, and then decreased over time. However, Eco did not induce an early release of d.s. DNA, but then extracellular DNA increased gradually, being maximal at 3 h. In line with this result, the release of extracellular DNA representing vital NETs in Pr or Ps-treated PMN was observed at 30 min in confocal microscopy experiments, where DNA was stained with PI (**Figure 7D**). To determine the origin of the DNA released in vital NETs, we performed qPCR using primers that amplify nuclear (actin) or mitochondrial (ND1) genes. The results shown in **Supplementary Figure 6** indicate that the DNA released in response to Pr and Ps is of nuclear origin. Altogether, the results presented indicate that Pr or Ps induce the early release of vital NETs that could be degraded by secreted bacterial DNases.

### Pr and Ps induce a low response at the inoculation site *in vivo* resulting in a rapid dissemination to organs

**Figure 8:**
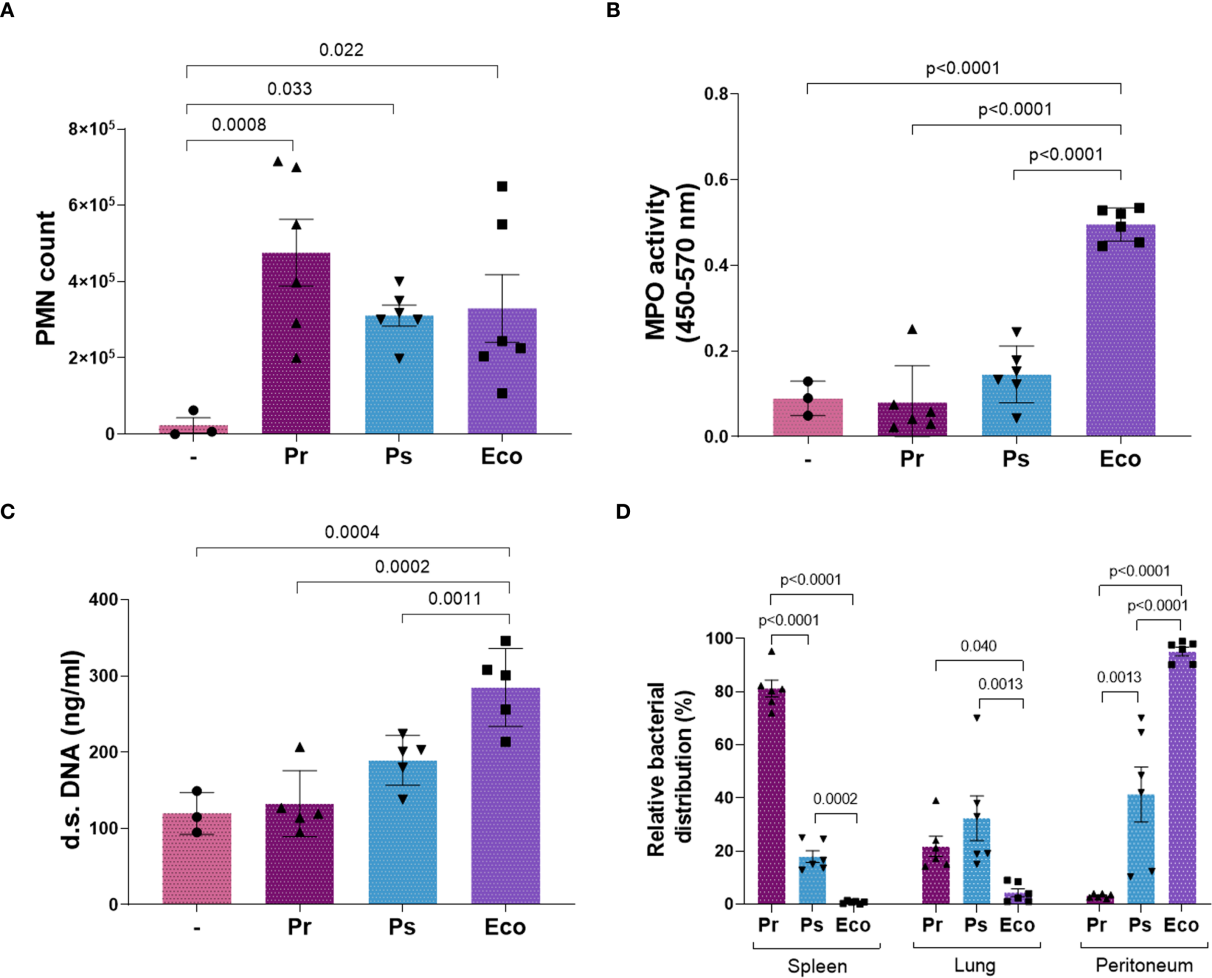
The lack of *in vivo* NETs formation after Pr and Ps infection increased bacterial dissemination to organs. Mice were inoculated i.p. with 1x10^6^ CFU of Pr, Ps, or Eco. After 24 h, the spleen, lungs, and the peritoneal liquid (pl) were collected. All results were expressed as the mean ± SEM; each point represents a different mouse. Data was analyzed using one-way ANOVA followed by Tukey´s post-test for multiple comparisons. **(A)** Number of PMN that migrated to the infectious focus (pl) in the presence of each bacterium. **(B)** MPO activity in the pl. **(C)** d.s. DNA in the pl. **(D)** Relative bacterial distribution (%) calculated as described in Materials and Methods by CFU determination in the pl, spleen, and lungs. Data was analyzed for each organ using one-way ANOVA followed by Tukey´s post-test for multiple comparisons.

To study the relevance of the evasion mechanisms described and validate our *in vitro* results, we inoculated Pr and Ps intraperitoneally (i.p.) in mice and used Eco for comparison. After 24 h, mice were sacrificed, and the peritoneal liquid (pl), the lungs, and spleen were obtained. As observed in **Figure 8**, the number of PMN that migrate to the pl was similar for Pr, Ps, and Eco (**Figure 8A**). However, only Eco increased MPO and the release of d.s. DNA in the pl, evidencing a local PMN activation and NETs formation (**Figures 8B, C**). In addition, to reveal the ability of Pr and Ps to escape the local PMN response, we studied the dissemination of bacteria by measuring the CFU present in the collected organs. As shown in **Figure 8D**, Pr was almost completely absent in the peritoneum and was found mostly in the spleen and to a minor extent in the lungs. Ps was distributed more evenly between the organs, showing an intermediate pattern, and Eco was mainly present in the peritoneum, being absent in the lungs and spleen. Altogether, these results indicate that Pr and Ps induce a poor PMN response at the primary infectious site, favoring their dissemination to other organs, whereas Eco triggers PMN activation and is contained at the inoculation site.

## Discussion

Antimicrobial resistance is one of the greatest global concerns in the 21^st^ century due to the rapid growth in the rate of this type of infection and the failure to develop new effective antimicrobial drugs at the needed speed. Besides the development of new drugs, the management of infections with multidrug-resistant microorganisms must be approached from different angles. Promoting the rational use of antimicrobials, maintaining strict measures of hygiene and control in hospitals, and monitoring outbreaks are fundamental pillars of this fight (1). Furthermore, studying the biology of infections with multidrug-resistant bacteria and their relationship with the immune system is essential to plan strategies that can contribute to the elimination of these types of pathogens. Opportunistic bacteria are microorganisms that normally do not cause disease in healthy individuals but can become pathogenic and cause infection in individuals with a weakened immune system due to underlying diseases, age, or those undergoing immunosuppressive therapy. One important characteristic of opportunistic bacteria is their ability to evade the immune system. Many pathogens have evolved different mechanisms to avoid detection and destruction by the host’s immune response. In this sense, the results presented in this work demonstrate that clinical selected isolates of *Providencia rettgeri* and *Providencia stuartii* are not the exception. To the best of our knowledge, our study explores for the first time whether PMN can recognize and eliminate these selected Pr and Ps isolates, revealing the presence of different evasion mechanisms that impact the PMN-bactericidal response, mainly affecting NETs, and favoring bacterial survival and dissemination. Since our study included only clinically relevant species of *Providencia* spp., our conclusions may be valid only for the selected isolates of Pr and Ps, and generalization to all *Providencia* spp. is not supported by the current isolate sampling. However, since some PMN activation parameters were also measured with other clinical isolates of Pr and Ps (CD11b, ROS and d.s. DNA release), yielding results similar to those obtained with the selected isolates, we can suggest that, at least for the parameters measured, what is reported here for the selected isolates could be valid for locally isolated Pr and Ps.

**Figure 9:**
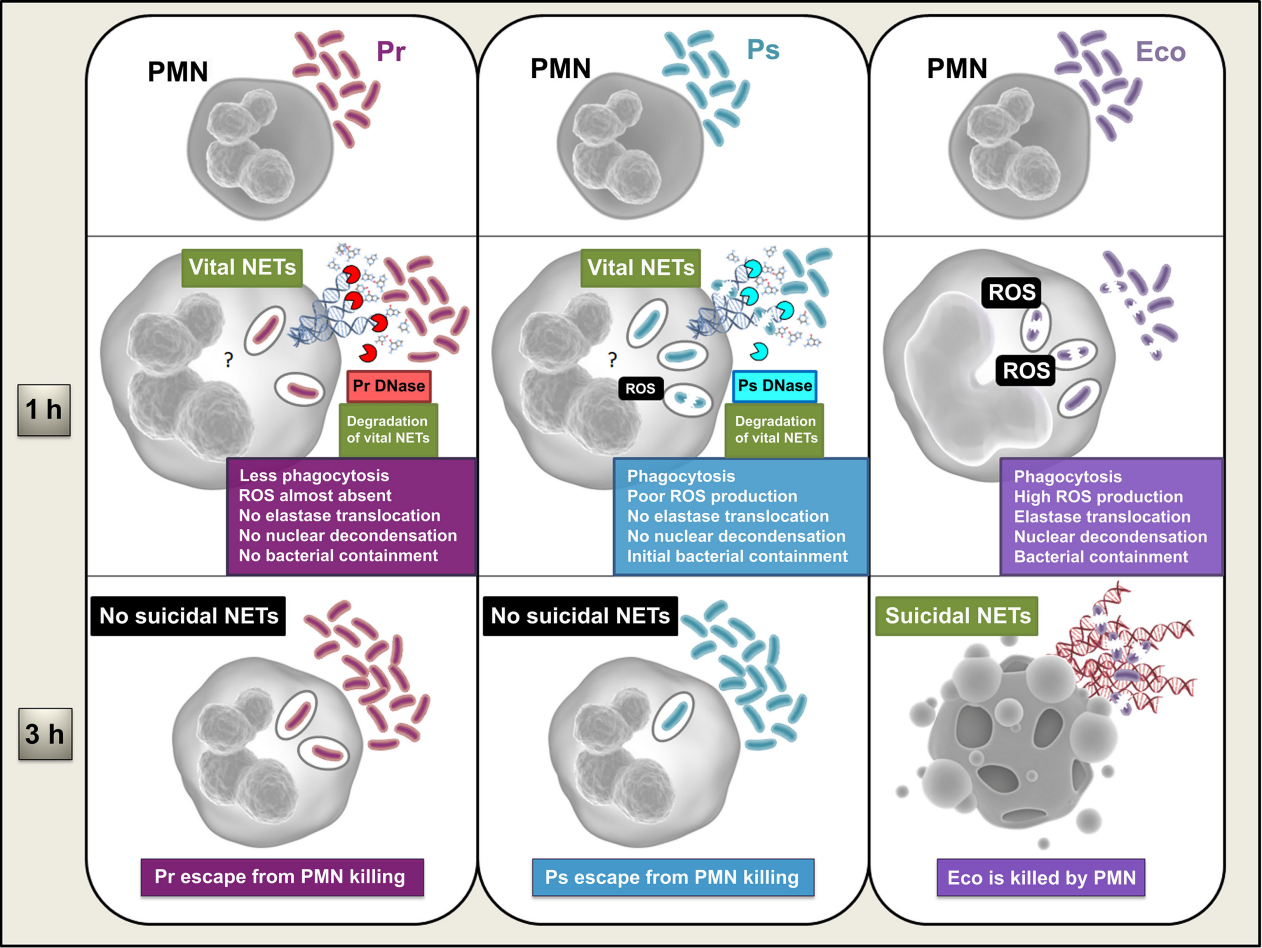
Schematic model summarizing the *in vitro* results obtained from this study. PMN become activated by Eco, phagocytosing the bacterium and inducing the production of ROS, a signal necessary for the early migration of elastase to the nucleus, DNA decondensation, and subsequent release of suicidal NETs, observed at 3 h. Full PMN activation results in the efficient elimination of Eco, involving both intracellular and extracellular bactericidal mechanisms of the PMN. Ps is also phagocytosed, but the generation of ROS induced in response to this phagocytosis is low. Whether the amount of ROS produced is insufficient, or because active evasion mechanisms mediated by the bacteria are taking place, neither elastase migration to the nucleus nor DNA decondensation is observed. However, the bacteria induce the production of vital NETs, which, combined with some Ps-induced PMN activation, generate an early partial containment of the bacteria. However, the release of a Ps-derived DNase degrades these vital NETs, which, together with the absence of suicide NETs induction, by an unknown mechanism (?), allow the bacteria to finally escape PMN-mediated death. Pr is also phagocytosed but the amount of internalization is lower, and ROS production is almost absent. Due to this low amount of ROS and/or due to some unexplored evasion mechanism mediated by the bacteria, no elastase migration to the nucleus or DNA decondensation is observed, and, consequently, no suicidal NETs are released. The mechanism by which Pr interferes with suicidal NETs formation is unknown (?). Similar to what was observed with Ps, Pr induces the formation of vital NETs and secretes DNases that degrade them. Pr induces less PMN activation in general and evades neutrophil bactericidal responses from an early stage, never being contained.

We have measured different PMN functions and compared the results obtained for the selected Pr and Ps isolates using an *E. coli* ATCC strain as a positive control, which is highly stimulatory for PMN, as previously reported (22, 23). A schematic model summarizing our *in vitro* results is shown in **Figure 9**. We found that *in vitro* the selected Pr and Ps were chemotactic and could be initially recognized by PMN, as demonstrated by increased FSC and CD11b up-regulation. Moreover, the percentage of phagocytosis was similar for Ps, Pr, and Eco, showing that the PMN’s early responses against bacteria were not affected. However, we found a higher number of PMN with 6 or 7 internalized Ps that could partially explain the higher ROS production observed for Ps compared to Pr, although both Ps and Pr induced a much lower respiratory burst compared to Eco. When we studied whether PMN could eliminate these Pr and Ps isolates, we found that Pr subverted PMN-mediated killing from the beginning, whereas Ps was initially contained at 1 h, but then increased its survival, also escaping PMN-mediated death at 3 h. The moderate but higher levels of ROS induced by Ps compared to Pr could also account for the differences observed in PMN-mediated killing at 1 h, where intracellular mechanisms may be more relevant for bacterial killing. Later, at 3 h, the formation of suicidal NETs contributes to extracellular-mediated killing, and at this point, neither Pr nor Ps induced this phenomenon. Therefore, although Ps appears to be initially controlled by PMN, its lack of subsequent restraint could be explained by the lack of suicidal NETs. Interestingly, we found that the isolates of Pr and Ps used in this study were inducers of vital NETs, but the release of DNases from bacteria may neutralize the bactericidal potential of this early induced mechanism.

It should be notice that the advantage of subverting NETs was also evidenced *in vivo*, where Eco induced PMN activation and local NETs release, and was contained at the inoculation site. However, no NETs were observed at the inoculation site for either Pr or Ps isolates used in our study, allowing bacteria to successfully and rapidly escape to other organs. The defensive role of PMN is unquestionable in bacterial infections, but the *in vivo* context includes other cells and molecules that could also be involved in bacterial elimination and have not been studied in this work. Nevertheless, our experiments in mice provide a global scenario, where the validity of our *in vitro* results is confirmed, regarding Pr and Ps ability to escape from PMN defensive mechanism.

An interesting finding was that the supernatant of both isolates of Pr and Ps showed a deoxyribonuclease (DNase) activity that could explain the decreased NETs formation observed in the presence of Pr or Ps together with PMA or Eco, although we cannot completely exclude the presence of other unknown bacterial-released factors that may also be influencing the ability of PMN to release NETs in response to other stimuli. Nevertheless, extracellular nucleases have been reported in several bacterial species, enabling them to escape from this immune trap and preventing the damage that antimicrobial proteins present in NETs could inflict (24, 25). This process not only contributes to their survival during an active immune response but can also enhance bacterial virulence by facilitating colonization and establishing infections.

When we discover the presence of DNases in the bacterial supernatant, we asked whether the absence of suicidal NETs could be related not to a lack of NETs formation but to their degradation. However, our experiments of nuclear decondensation indicated that the early mechanisms necessary for a posterior suicidal NETs release were not occurring with the Pr or Ps isolates used. In this sense, degradation of NETs by the secreted DNases may not be the central mechanism explaining the lack of suicidal NETs induction and another unexplored mechanism may be involved. However, considering that vital NETs are induced by the selected Pr and Ps isolates, DNase may be involved in the degradation of this type of NETs. Moreover, bacterial-derived DNases may also favor the dissemination of other NET-inducer microorganisms in the context of co-infections, which are common in hospitalized patients (26, 27). In this sense, during the COVID-19 pandemic, a high number of SARS-CoV-2 patients co-infected with gram-negative microorganisms were reported, generally increasing the risk of serious illness (28, 29). Besides their role in NETs degradation, bacteria-secreted DNases are also important in other processes, such as microbial competition or nutrient cycling. Degradation of extracellular bacterial DNA within the microbial environment reduces the viscosity of biofilms and promotes the dispersal of bacterial cells (30). Moreover, these enzymes contribute to the turnover of nucleic acids, which can be repurposed as a nutrient source (31). Therefore, our findings are also important for a comprehensive understanding of microbial ecology.

Our study sheds light on the interaction of the innate immune system with the selected Pr and Ps isolates, two multidrug-resistant bacteria of increasing clinical relevance. We are demonstrating the existence of different immune evasion mechanisms in these bacteria, affecting the generation of a central PMN microbicidal mechanism for bacterial containment and elimination, NETs formation. It is essential to understand host-pathogen interactions and immune evasion mechanisms to plan more effective antibacterial therapies aimed at unblocking evasion mechanisms, making the immune system more efficient in the battle against multi-resistant bacteria.

## Data Availability

Datasets are available on request: The raw data supporting the conclusions of this article will be made available by the authors, without undue reservation. The isolates M21250 and M17517 have been submitted to GenBank under de BioProject PRJNA1304938.
