# Supplementary figures and images for: Clinical isolates of Providencia rettgeri and Providencia Stuartii evades neutrophil-mediated killing by subverting neutrophil-extracellular traps
Source: Front Immunol. 2025 Oct 2;16:1636387. doi: 10.3389/fimmu.2025.1636387 (PMC12528210; doi:10.3389/fimmu.2025.1636387)

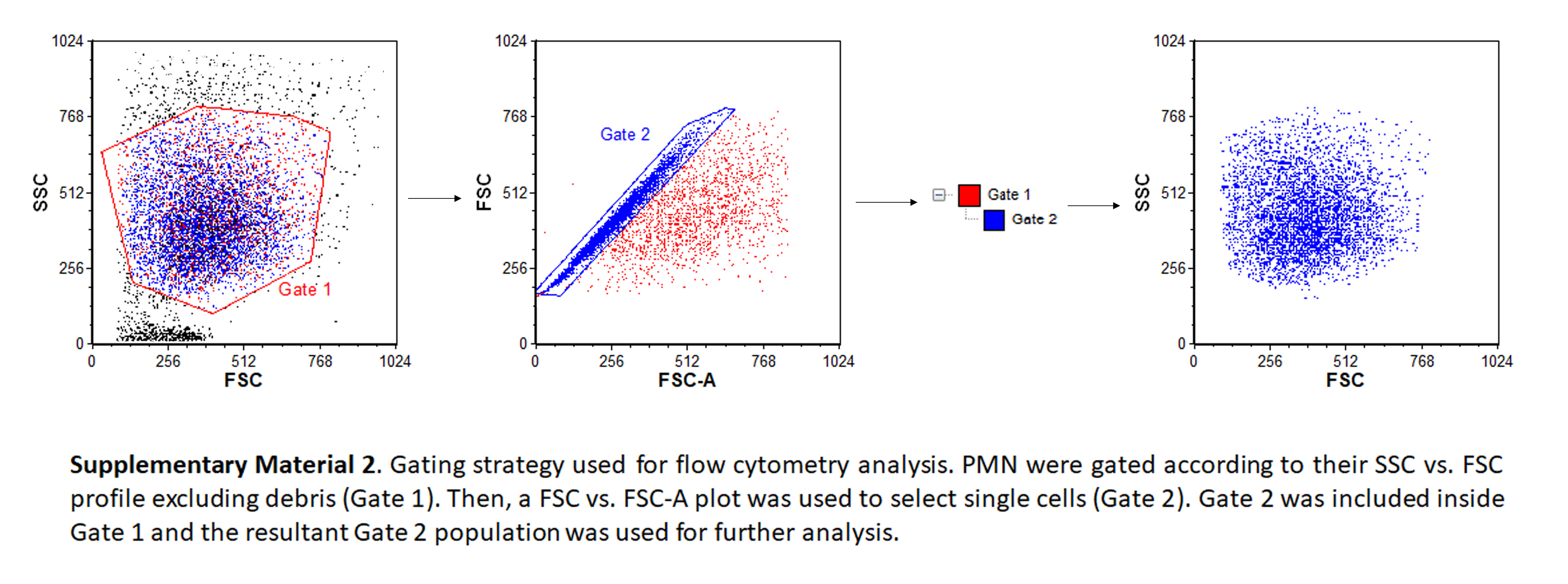

Supplement: Supplementary file 1 [file Image1.tif]

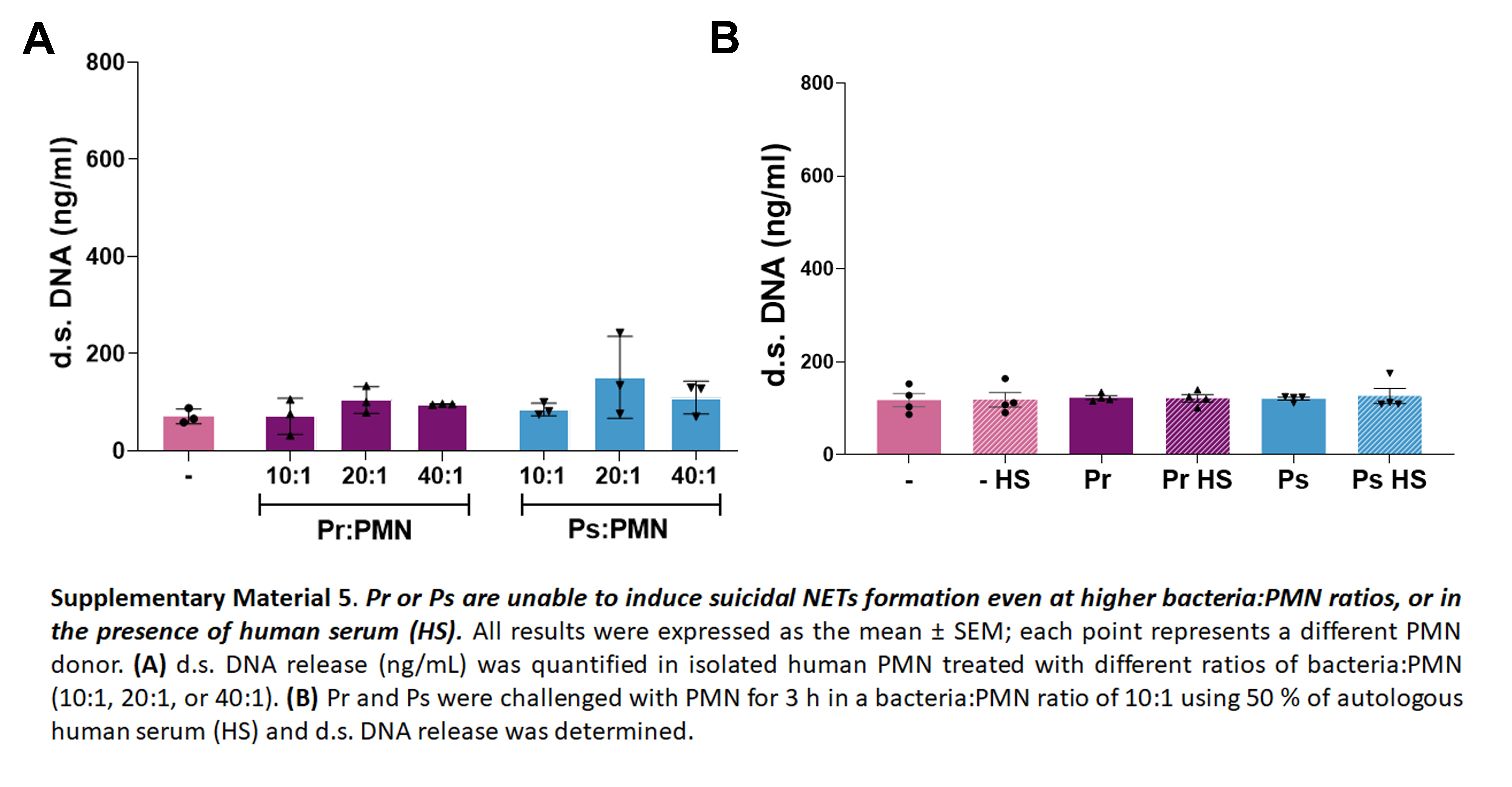

Supplement: Supplementary file 3 [file Image3.tif]

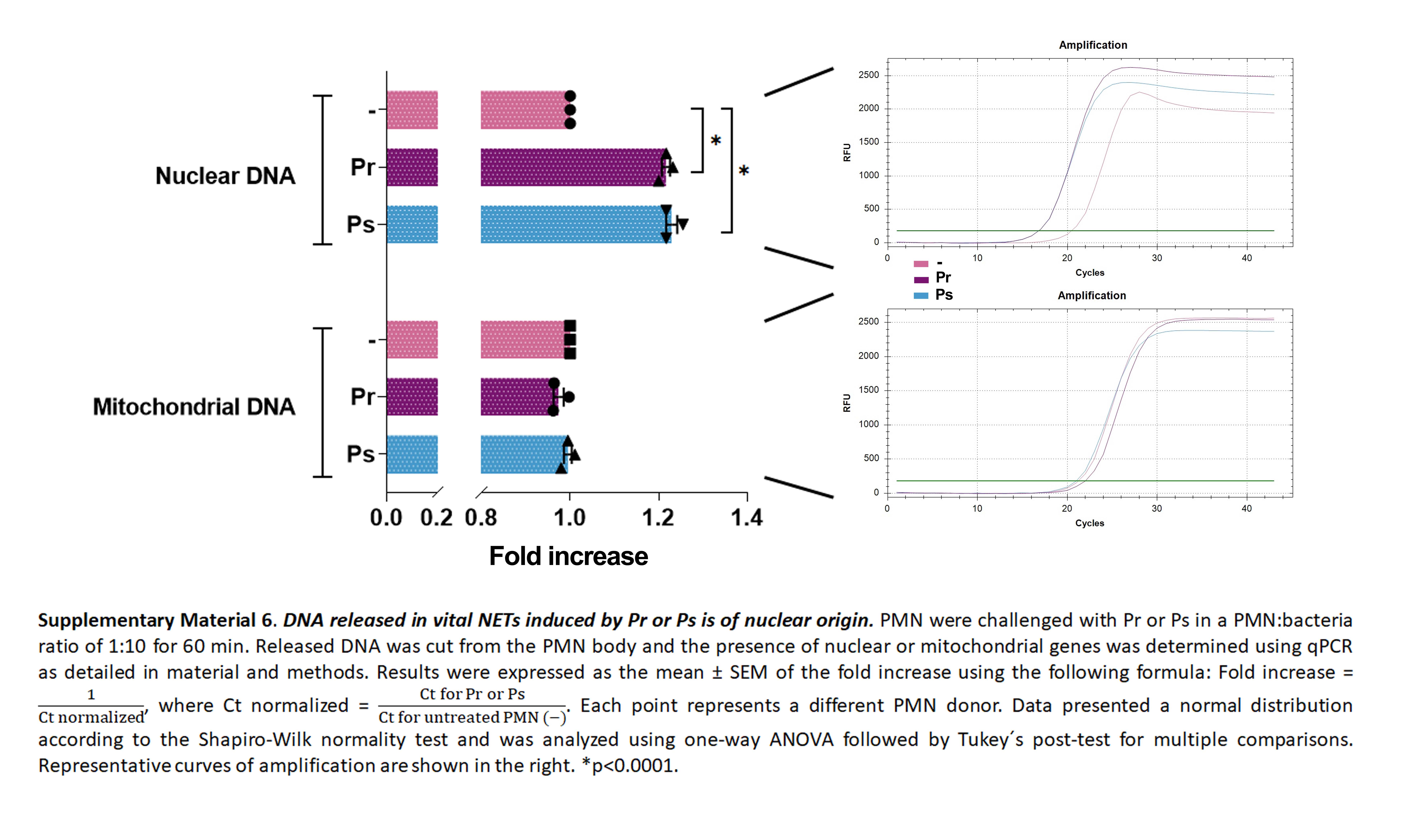

Supplement: Supplementary file 4 [file Image4.tif]
