# Supplementary material for: Clinical isolates of Providencia rettgeri and Providencia Stuartii evades neutrophil-mediated killing by subverting neutrophil-extracellular traps
Source: Front Immunol. 2025 Oct 2;16:1636387. doi: 10.3389/fimmu.2025.1636387 (PMC12528210; doi:10.3389/fimmu.2025.1636387)
Supplement: Supplementary file 5 [file DataSheet1.pdf]

Supplementary material 3. Statistical analysis

Figure 1. CFU

ANOVA size effect (η^2)= 0,74

Tukey's multiple comparisons test	Mean Diff,	95,00% CI of diff,	Significant?	Summary	P Value
Ps 1 vs. Pr 1	-0,4244	-0,6182 to -0,2307	Yes	****	<0,0001
Ps 1 vs. Eco 1	0,2978	0,1040 to 0,4915	Yes	**	0,0022
Pr 1 vs. Eco 1	0,7222	0,5285 to 0,9160	Yes	****	<0,0001
Ps 3 vs. Pr 3	-0,03714	-0,6149 to 0,5406	No	ns	0,9853
Ps 3 vs. Eco 3	1,253	0,6751 to 1,831	Yes	****	<0,0001
Pr 3 vs. Eco 3	1,29	0,8051 to 1,775	Yes	****	<0,0001
Ps 1 vs. Ps 3	-0,7802	-1,237 to -0,3230	Yes	***	0,0001
Pr 1 vs. Pr 3	-0,3929	-0,8500 to 0,06429	No	ns	0,1288
Eco 1 vs. Eco 3	0,1749	-0,2822 to 0,6321	No	ns	0,8608

Figure 2A. Qtx

ANOVA size effect (η^2)= 0,95

Tukey's multiple comparisons test	Mean Diff,	95,00% CI of diff,	Significant?	Summary	P Value
- vs. Ps	-18,83	-24,81 to -12,85	Yes	****	<0,0001
- vs. Pr	-27,83	-33,81 to -21,85	Yes	****	<0,0001
- vs. Eco	-43,83	-49,81 to -37,85	Yes	****	<0,0001
Ps vs. Pr	-9	-14,98 to -3,021	Yes	**	0,0022
Ps vs. Eco	-25	-30,98 to -19,02	Yes	****	<0,0001
Pr vs. Eco	-16	-21,98 to -10,02	Yes	****	<0,0001

Figure 2B. FSC

ANOVA size effect (η^2)= 0,91

Tukey's multiple comparisons test	Mean Diff,	95,00% CI of diff,	Significant?	Summary	P Value
- vs. Pr	-43,78	-55,21 to -32,35	Yes	****	<0,0001
- vs. Ps	-44,12	-55,55 to -32,69	Yes	****	<0,0001
- vs. Eco	-51,57	-63,00 to -40,14	Yes	****	<0,0001
Pr vs. Ps	-0,3333	-11,76 to 11,10	No	ns	0,9998
Pr vs. Eco	-7,783	-19,21 to 3,647	No	ns	0,2571
Ps vs. Eco	-7,45	-18,88 to 3,980	No	ns	0,2916

Figure 2C. CD11b

ANOVA size effect (η^2)= 0,88

Tukey's multiple comparisons test	Mean Diff,	95,00% CI of diff,	Significant?	Summary	P Value
- vs. Ps	-87,4	-174,5 to -0,3037	Yes	*	0,049
- vs. Pr	-158,9	-246,0 to -71,82	Yes	***	0,0003
- vs. Eco	-365,4	-452,4 to -278,3	Yes	****	<0,0001
Ps vs. Pr	-71,52	-158,6 to 15,57	No	ns	0,1319
Ps vs. Eco	-278	-365,1 to -190,9	Yes	****	<0,0001
Pr vs. Eco	-206,4	-293,5 to -119,3	Yes	****	<0,0001

Figure 3A. % Phagocytosis

ANOVA size effect (η^2)=

Tukey's multiple comparisons test	Mean Diff,	95,00% CI of diff,	Significant?	Summary	P Value
Ps vs. Pr	8,183	-16,74 to 33,11	No	ns	0,6331
Ps vs. Eco	19,4	-7,518 to 46,32	No	ns	0,1602
Pr vs. Eco	11,22	-15,70 to 38,14	No	ns	0,4906

Figure 3C. % ROS

ANOVA size effect (η^2)= 0,99

Tukey's multiple comparisons test	Mean Diff,	95,00% CI of diff,	Significant?	Summary	Adjusted P Value
- vs. Ps	-22,54	-26,61 to -18,48	Yes	****	<0,0001
- vs. Pr	-7,492	-11,56 to -3,425	Yes	***	0,0003
- vs. Eco	-63,27	-67,33 to -59,20	Yes	****	<0,0001
Ps vs. Pr	15,05	10,99 to 19,12	Yes	****	<0,0001
Ps vs. Eco	-40,73	-44,79 to -36,66	Yes	****	<0,0001
Pr vs. Eco	-55,78	-59,84 to -51,71	Yes	****	<0,0001

Figure 3D. MFI ROS

ANOVA size effect (η^2)= 0,84

Tukey's multiple comparisons test	Mean Diff,	95,00% CI of diff,	Significant?	Summary	P Value
- vs. Pr	0,04	-116,2 to 116,3	No	ns	>0,9999
- vs. Ps	-121,4	-237,6 to -5,152	Yes	*	0,0387
- vs. Eco	-277,1	-393,3 to -160,8	Yes	****	<0,0001
Pr vs. Ps	-121,4	-237,7 to -5,192	Yes	*	0,0386
Pr vs. Eco	-277,1	-393,3 to -160,8	Yes	****	<0,0001
Ps vs. Eco	-155,7	-271,9 to -39,41	Yes	**	0,0064

Figure 4A. NETs area

ANOVA size effect (η^2)= 0,93

Tukey's multiple comparisons test	Mean Diff,	95,00% CI of diff,	Significant?	Summary	P Value
- vs. Ps	1092	-5239 to 7423	No	ns	0,9546
- vs. Pr	224,3	-6107 to 6555	No	ns	0,9996
- vs. Eco	-21412	-27743 to -15081	Yes	****	<0,0001
Ps vs. Pr	-867,8	-7199 to 5463	No	ns	0,9762
Ps vs. Eco	-22504	-28835 to -16173	Yes	****	<0,0001
Pr vs. Eco	-21636	-27967 to -15305	Yes	****	<0,0001

Figure 4B. ds DNA

ANOVA size effect (η^2)= 0,97

Tukey's multiple comparisons test	Mean Diff,	95,00% CI of diff,	Significant?	Summary	P Value
- vs. Pr	-7,067	-55,04 to 40,91	No	ns	0,9757
- vs. Ps	-42,25	-90,22 to 5,722	No	ns	0,0967
- vs. Eco	-377,9	-425,8 to -329,9	Yes	****	<0,0001
Pr vs. Ps	-35,18	-83,16 to 12,79	No	ns	0,2027

Pr vs. Eco	-370,8	-418,8 to -322,8	Yes	****	<0,0001
Ps vs. Eco	-335,6	-383,6 to -287,6	Yes	****	<0,0001

Figure 4C. PI

ANOVA size effect (η^2)= 0,98

Tukey's multiple comparisons test	Mean Diff,	95,00% CI of diff,	Significant?	Summary	P Value
- vs. Pr	2,9	-4,553 to 10,35	No	ns	0,6644
- vs. Ps	0,4875	-6,965 to 7,940	No	ns	0,9973
- vs. Eco	-51,98	-59,43 to -44,52	Yes	****	<0,0001
Pr vs. Ps	-2,413	-9,865 to 5,040	No	ns	0,7733
Pr vs. Eco	-54,88	-62,33 to -47,42	Yes	****	<0,0001
Ps vs. Eco	-52,46	-59,92 to -45,01	Yes	****	<0,0001

Figure 5A. Area NETs + Eco

ANOVA size effect (η^2)= 0,88

Tukey's multiple comparisons test	Mean Diff,	95,00% CI of diff,	Significant?	Summary	P Value
- vs. Ps	1092	-6782 to 8966	No	ns	0,9975
- vs. Pr	224,3	-7650 to 8098	No	ns	>0,9999
- vs. Eco	-22162	-30036 to -14288	Yes	****	<0,0001
- vs. Eco+Ps	-2134	-10008 to 5740	No	ns	0,951
- vs. Eco+Pr	57,25	-7817 to 7931	No	ns	>0,9999
Ps vs. Pr	-867,8	-8742 to 7006	No	ns	0,9992
Ps vs. Eco	-23254	-31128 to -15380	Yes	****	<0,0001
Ps vs. Eco+Ps	-3226	-11100 to 4648	No	ns	0,7803
Ps vs. Eco+Pr	-1035	-8909 to 6839	No	ns	0,9981
Pr vs. Eco	-22386	-30260 to -14512	Yes	****	<0,0001
Pr vs. Eco+Ps	-2358	-10232 to 5516	No	ns	0,9271
Pr vs. Eco+Pr	-167	-8041 to 7707	No	ns	>0,9999
Eco vs. Eco+Ps	20028	12154 to 27902	Yes	****	<0,0001
Eco vs. Eco+Pr	22219	14345 to 30093	Yes	****	<0,0001
Eco+Ps vs. Eco+Pr	2191	-5683 to 10065	No	ns	0,9455

Figure 5B. ds DNA + Eco

ANOVA size effect (η^2)= 0,87

Tukey's multiple comparisons test	Mean Diff,	95,00% CI of diff,	Significant?	Summary	P Value
- vs. Ps	-9,575	-110,6 to 91,49	No	ns	0,9996
- vs. Pr	-11,98	-113,0 to 89,09	No	ns	0,9988
- vs. Eco	-285,3	-386,4 to -184,3	Yes	****	<0,0001
- vs. Eco+Ps	-143,2	-244,2 to -42,09	Yes	**	0,0032
- vs. Eco+Pr	-133	-234,0 to -31,91	Yes	**	0,0063
Ps vs. Pr	-2,4	-103,5 to 98,66	No	ns	>0,9999
Ps vs. Eco	-275,8	-376,8 to -174,7	Yes	****	<0,0001
Ps vs. Eco+Ps	-133,6	-234,6 to -32,51	Yes	**	0,006
Ps vs. Eco+Pr	-123,4	-224,5 to -22,34	Yes	*	0,0119

Pr vs. Eco	-273,4	-374,4 to -172,3	Yes	****	<0,0001
Pr vs. Eco+Ps	-131,2	-232,2 to -30,11	Yes	**	0,0071
Pr vs. Eco+Pr	-121	-222,1 to -19,94	Yes	*	0,0139
Eco vs. Eco+Ps	142,2	41,11 to 243,2	Yes	**	0,0034
Eco vs. Eco+Pr	152,4	51,29 to 253,4	Yes	**	0,0017
Eco+Ps vs. Eco+Pr	10,18	-90,89 to 111,2	No	ns	0,9995

Figure 5C. Area NETs + PMA

ANOVA size effect (η^2)= 0,86

Tukey's multiple comparisons test	Mean Diff,	95,00% CI of diff,	Significant?	Summary	P Value
- vs. Ps	877,7	-18704 to 20460	No	ns	>0,9999
- vs. Pr	-2884	-22466 to 16698	No	ns	0,9954
- vs. PMA	-68008	-87590 to -48426	Yes	****	<0,0001
- vs. PMA+Ps	-15916	-35498 to 3666	No	ns	0,1395
- vs. PMA+Pr	-9759	-29341 to 9823	No	ns	0,571
Ps vs. Pr	-3762	-23344 to 15820	No	ns	0,9848
Ps vs. PMA	-68885	-88467 to -49303	Yes	****	<0,0001
Ps vs. PMA+Ps	-16794	-36376 to 2788	No	ns	0,1098
Ps vs. PMA+Pr	-10637	-30219 to 8945	No	ns	0,4867
Pr vs. PMA	-65123	-84705 to -45541	Yes	****	<0,0001
Pr vs. PMA+Ps	-13032	-32614 to 6550	No	ns	0,2904
Pr vs. PMA+Pr	-6875	-26457 to 12707	No	ns	0,8384
PMA vs. PMA+Ps	52091	32509 to 71673	Yes	****	<0,0001
PMA vs. PMA+Pr	58249	38667 to 77831	Yes	****	<0,0001
PMA+Ps vs. PMA+Pr	6157	-13425 to 25739	No	ns	0,8893

Figure 5D. ds DNA + PMA

ANOVA size effect (η^2)= 0,94

Tukey's multiple comparisons test	Mean Diff,	95,00% CI of diff,	Significant?	Summary	P Value
- vs. Ps	-66,23	-166,5 to 34,01	No	ns	0,3608
- vs. Pr	-99,58	-199,8 to 0,6627	No	ns	0,0523
- vs. PMA	-407,8	-508,1 to -307,6	Yes	****	<0,0001
- vs. PMA+Ps	-132	-232,2 to -31,72	Yes	**	0,0046
- vs. PMA+Pr	-145,7	-246,0 to -45,49	Yes	**	0,0015
Ps vs. Pr	-33,35	-133,6 to 66,89	No	ns	0,9103
Ps vs. PMA	-341,6	-441,9 to -241,4	Yes	****	<0,0001
Ps vs. PMA+Ps	-65,73	-166,0 to 34,51	No	ns	0,3688
Ps vs. PMA+Pr	-79,5	-179,7 to 20,74	No	ns	0,1842
Pr vs. PMA	-308,3	-408,5 to -208,0	Yes	****	<0,0001
Pr vs. PMA+Ps	-32,38	-132,6 to 67,85	No	ns	0,9199
Pr vs. PMA+Pr	-46,15	-146,4 to 54,09	No	ns	0,7265
PMA vs. PMA+Ps	275,9	175,6 to 376,1	Yes	****	<0,0001
PMA vs. PMA+Pr	262,1	161,9 to 362,4	Yes	****	<0,0001
PMA+Ps vs. PMA+Pr	-13,77	-114,0 to 86,47	No	ns	0,9982

Figure 6A. ds DNA - bact.(PFA)

ANOVA size effect (η2)= 0,98

Tukey's multiple comparisons test	Mean Diff,	95,00% CI of diff,	Significant?	Summary	P Value
- vs. Ps (PFA)	-13,75	-49,18 to 21,68	No	ns	0,8152
- vs. Pr (PFA)	-6	-41,43 to 29,43	No	ns	0,9937
- vs. PMA	-193,5	-228,9 to -158,1	Yes	****	<0,0001
- vs. PMA+Ps (PFA)	-180,8	-216,2 to -145,3	Yes	****	<0,0001
- vs. PMA+Pr (PFA)	-193,3	-228,7 to -157,8	Yes	****	<0,0001
Ps (PFA) vs. Pr (PFA)	7,75	-27,68 to 43,18	No	ns	0,9802
Ps (PFA) vs. PMA	-179,8	-215,2 to -144,3	Yes	****	<0,0001
Ps (PFA) vs. PMA+Ps (PFA)	-167	-202,4 to -131,6	Yes	****	<0,0001
Ps (PFA) vs. PMA+Pr (PFA)	-179,5	-214,9 to -144,1	Yes	****	<0,0001
Pr (PFA) vs. PMA	-187,5	-222,9 to -152,1	Yes	****	<0,0001
Pr (PFA) vs. PMA+Ps (PFA)	-174,8	-210,2 to -139,3	Yes	****	<0,0001
Pr (PFA) vs. PMA+Pr (PFA)	-187,3	-222,7 to -151,8	Yes	****	<0,0001
PMA vs. PMA+Ps (PFA)	12,75	-22,68 to 48,18	No	ns	0,8568
PMA vs. PMA+Pr (PFA)	0,25	-35,18 to 35,68	No	ns	>0,9999
PMA+Ps (PFA) vs. PMA+Pr (PFA)	-12,5	-47,93 to 22,93	No	ns	0,8663

Figure 6B. ds DNA - bact. (sn)

ANOVA size effect (η2)= 0,72

Tukey's multiple comparisons test	Mean Diff,	95,00% CI of diff,	Significant?	Summary	P Value
- vs. sn Ps	-30,4	-127,1 to 66,25	No	ns	0,9222
- vs. sn Pr	-24,6	-121,3 to 72,05	No	ns	0,9671
- vs. PMA	-211,4	-308,1 to -114,7	Yes	****	<0,0001
- vs. PMA+sn Ps	-108,4	-205,1 to -11,75	Yes	*	0,0217
- vs. PMA+sn Pr	-93,2	-189,9 to 3,450	No	ns	0,0632
sn Ps vs. sn Pr	5,8	-90,85 to 102,5	No	ns	>0,9999
sn Ps vs. PMA	-181	-277,7 to -84,35	Yes	****	<0,0001
sn Ps vs. PMA+sn Ps	-78	-174,7 to 18,65	No	ns	0,1651
sn Ps vs. PMA+sn Pr	-62,8	-159,5 to 33,85	No	ns	0,3665
sn Pr vs. PMA	-186,8	-283,5 to -90,15	Yes	****	<0,0001
sn Pr vs. PMA+sn Ps	-83,8	-180,5 to 12,85	No	ns	0,1163
sn Pr vs. PMA+sn Pr	-68,6	-165,3 to 28,05	No	ns	0,2766
PMA vs. PMA+sn Ps	103	6,350 to 199,7	Yes	*	0,032
PMA vs. PMA+sn Pr	118,2	21,55 to 214,9	Yes	*	0,0104
PMA+sn Ps vs. PMA+sn Pr	15,2	-81,45 to 111,9	No	ns	0,9962

Figure 6C. ds DNA - eukaryotic DNA

ANOVA size effect (η2)= 0,94

Tukey's multiple comparisons test	Mean Diff,	95,00% CI of diff,	Significant?	Summary	P Value
- vs. sn Ps	506,3	306,0 to 706,7	Yes	****	<0,0001
- vs. sn Pr	554	353,7 to 754,3	Yes	****	<0,0001

- vs. sn Ps+EDTA	120	-80,35 to 320,3	No	ns	0,4311
- vs. sn Pr+EDTA	48,67	-151,7 to 249,0	No	ns	0,9774
- vs. Nuc S7	575	374,7 to 775,3	Yes	****	<0,0001
- vs. Nuc S7+EDTA	126,3	-74,01 to 326,7	No	ns	0,376
sn Ps vs. sn Pr	47,67	-152,7 to 248,0	No	ns	0,9796
sn Ps vs. sn Ps+EDTA	-386,3	-586,7 to -186,0	Yes	***	0,0002
sn Ps vs. sn Pr+EDTA	-457,7	-658,0 to -257,3	Yes	****	<0,0001
sn Ps vs. Nuc S7	68,67	-131,7 to 269,0	No	ns	0,8941
sn Ps vs. Nuc S7+EDTA	-380	-580,3 to -179,7	Yes	***	0,0002
sn Pr vs. sn Ps+EDTA	-434	-634,3 to -233,7	Yes	****	<0,0001
sn Pr vs. sn Pr+EDTA	-505,3	-705,7 to -305,0	Yes	****	<0,0001
sn Pr vs. Nuc S7	21	-179,3 to 221,3	No	ns	0,9998
sn Pr vs. Nuc S7+EDTA	-427,7	-628,0 to -227,3	Yes	****	<0,0001
sn Ps+EDTA vs. sn Pr+EDTA	-71,33	-271,7 to 129,0	No	ns	0,8768
sn Ps+EDTA vs. Nuc S7	455	254,7 to 655,3	Yes	****	<0,0001
sn Ps+EDTA vs. Nuc S7+EDTA	6,333	-194,0 to 206,7	No	ns	>0,9999
sn Pr+EDTA vs. Nuc S7	526,3	326,0 to 726,7	Yes	****	<0,0001
sn Pr+EDTA vs. Nuc S7+EDTA	77,67	-122,7 to 278,0	No	ns	0,8304
Nuc S7 vs. Nuc S7+EDTA	-448,7	-649,0 to -248,3	Yes	****	<0,0001

Figure 7A. DNA decondensation

ANOVA size effect (η2)= 0,90

Tukey's multiple comparisons test	Mean Diff,	95,00% CI of diff,	Significant?	Summary	P Value
- vs. Ps	-2,577	-24,34 to 19,19	No	ns	0,9965
- vs. Pr	-16,07	-37,83 to 5,701	No	ns	0,2203
- vs. Eco	-66,03	-86,79 to -45,28	Yes	****	<0,0001
- vs. PMA	-78,43	-100,2 to -56,66	Yes	****	<0,0001
Ps vs. Pr	-13,49	-36,22 to 9,246	No	ns	0,4203
Ps vs. Eco	-63,45	-85,22 to -41,69	Yes	****	<0,0001
Ps vs. PMA	-75,85	-98,59 to -53,12	Yes	****	<0,0001
Pr vs. Eco	-49,97	-71,73 to -28,20	Yes	****	<0,0001
Pr vs. PMA	-62,37	-85,10 to -39,63	Yes	****	<0,0001
Eco vs. PMA	-12,4	-34,17 to 9,367	No	ns	0,4599

Figure 7C. Cinetica DNA

0,5 h. ANOVA size effect (η2)= 0,80

Tukey's multiple comparisons test	Mean Diff,	95,00% CI of diff,	Significant?	Summary	P Value
Pr 0.5 vs. Ps 0.5	2,18	0,05126 to 4,310	Yes	*	0,045
Pr 0.5 vs. Eco 0.5	4,642	2,513 to 6,772	Yes	***	0,0005
Ps 0.5 vs. Eco 0.5	2,462	0,3329 to 4,591	Yes	*	0,0253

1 h. ANOVA size effect (η2)= 0,87

Tukey's multiple comparisons test	Mean Diff,	95,00% CI of diff,	Significant?	Summary	P Value
Pr 1 vs. Ps 1	-0,4003	-1,691 to 0,8903	No	ns	0,6737

Pr 1 vs. Eco 1	2,976	1,686 to 4,267	Yes	***	0,0003
Ps 1 vs. Eco 1	3,377	2,086 to 4,667	Yes	***	0,0001

3 h. ANOVA size effect (η2)= 0,85

Tukey's multiple comparisons test	Mean Diff,	95,00% CI of diff,	Significant?	Summary	P Value
Pr 3 vs. Ps 3	-0,3834	-1,399 to 0,6324	No	ns	0,6109
Pr 3 vs. Eco 3	-4,25	-5,347 to -3,153	Yes	****	<0,0001
Ps 3 vs. Eco 3	-3,867	-4,964 to -2,770	Yes	****	<0,0001

Figure 8A. PMN count in the pl

ANOVA size effect (η2)= 0,54

Tukey's multiple comparisons test	Mean Diff,	95,00% CI of diff,	Significant?	Summary	P Value
- vs. Ps	-292256	-565536 to -18976	Yes	*	0,0335
- vs. Pr	-457256	-730536 to -183976	Yes	***	0,0008
- vs. Eco	-311256	-584536 to -37976	Yes	*	0,0222
Ps vs. Pr	-165000	-425562 to 95562	No	ns	0,3127
Ps vs. Eco	-19000	-279562 to 241562	No	ns	0,9968
Pr vs. Eco	146000	-114562 to 406562	No	ns	0,4152

Figure 8B. MPO activity in the pl

ANOVA size effect (η2)= 0,90

Tukey's multiple comparisons test	Mean Diff,	95,00% CI of diff,	Significant?	Summary	P Value
- vs. Ps	-0,056	-0,1849 to 0,07286	No	ns	0,614
- vs. Pr	0,009833	-0,1190 to 0,1387	No	ns	0,9963
- vs. Eco	-0,4057	-0,5345 to -0,2768	Yes	****	<0,0001
Ps vs. Pr	0,06583	-0,03938 to 0,1710	No	ns	0,3168
Ps vs. Eco	-0,3497	-0,4549 to -0,2445	Yes	****	<0,0001
Pr vs. Eco	-0,4155	-0,5207 to -0,3103	Yes	****	<0,0001

Figure 8C. d.s DNA in the pl

ANOVA size effect (η2)= 0,76

Tukey's multiple comparisons test	Mean Diff,	95,00% CI of diff,	Significant?	Summary	P Value
- vs. Ps	-69,6	-157,0 to 17,78	No	ns	0,1416
- vs. Pr	-12,8	-100,2 to 74,58	No	ns	0,9731
- vs. Eco	-165,2	-252,6 to -77,84	Yes	***	0,0004
Ps vs. Pr	56,8	-18,87 to 132,5	No	ns	0,1762
Ps vs. Eco	-95,62	-171,3 to -19,95	Yes	*	0,0119
Pr vs. Eco	-152,4	-228,1 to -76,75	Yes	***	0,0002

Figure 8D. Relative distribution (%)

Spleen. ANOVA size effect (η2)= 0,98

Tukey's multiple comparisons test	Mean Diff,	95,00% CI of diff,	Significant?	Summary	P Value
Pr vs. Ps	63,29	55,00 to 71,57	Yes	****	<0,0001
Pr vs. Eco	80,49	72,20 to 88,78	Yes	****	<0,0001

Ps vs. Eco	17,21	8,920 to 25,49	Yes	***	0,0002

Lung. ANOVA size effect (η2)= 0,50

Tukey's multiple comparisons test	Mean Diff,	95,00% CI of diff,	Significant?	Summary	P Value
Pr vs. Ps	-10,35	-26,80 to 6,107	No	ns	0,2692
Pr vs. Eco	17,03	0,5768 to 33,49	Yes	*	0,0418
Ps vs. Eco	27,38	10,92 to 43,83	Yes	**	0,0013

Pl. ANOVA size effect (η2)= 0,88

Tukey's multiple comparisons test	Mean Diff,	95,00% CI of diff,	Significant?	Summary	P Value
Pr vs. Ps	-38,17	-60,42 to -15,92	Yes	**	0,0013
Pr vs. Eco	-92,05	-114,3 to -69,80	Yes	****	<0,0001
Ps vs. Eco	-53,88	-76,13 to -31,63	Yes	****	<0,0001
